# Capsules, Toxins and AtxA as Virulence Factors of Emerging *Bacillus cereus* Biovar *anthracis*

**DOI:** 10.1371/journal.pntd.0003455

**Published:** 2015-04-01

**Authors:** Christophe Brézillon, Michel Haustant, Susann Dupke, Jean-Philippe Corre, Angelika Lander, Tatjana Franz, Marc Monot, Evelyne Couture-Tosi, Gregory Jouvion, Fabian H. Leendertz, Roland Grunow, Michèle E. Mock, Silke R. Klee, Pierre L. Goossens

**Affiliations:** 1 Institut Pasteur, Pathogénie des Toxi-Infections Bactériennes, Paris, France; 2 Robert Koch-Institut, Centre for Biological Threats and Special Pathogens (ZBS 2), Berlin, Germany; 3 Institut Pasteur, Pathogenèse des bactéries anaérobies, Paris, France; 4 Institut Pasteur, Unité Histopathologie Humaine et Modèles Animaux, Paris, France; 5 Robert Koch-Institut, Epidemiology of Highly Pathogenic Microorganisms (P 3), Berlin, Germany; University of Tennessee, UNITED STATES

## Abstract

Emerging *B. cereus* strains that cause anthrax-like disease have been isolated in Cameroon (CA strain) and Côte d’Ivoire (CI strain). These strains are unusual, because their genomic characterisation shows that they belong to the *B. cereus* species, although they harbour two plasmids, pBCXO1 and pBCXO2, that are highly similar to the pXO1 and pXO2 plasmids of *B. anthracis* that encode the toxins and the polyglutamate capsule respectively. The virulence factors implicated in the pathogenicity of these *B. cereus* bv *anthracis* strains remain to be characterised. We tested their virulence by cutaneous and intranasal delivery in mice and guinea pigs; they were as virulent as wild-type *B. anthracis*. Unlike as described for pXO2-cured *B. anthracis*, the CA strain cured of the pBCXO2 plasmid was still highly virulent, showing the existence of other virulence factors. Indeed, these strains concomitantly expressed a hyaluronic acid (HA) capsule and the *B. anthracis* polyglutamate (PDGA) capsule. The HA capsule was encoded by the *hasACB* operon on pBCXO1, and its expression was regulated by the global transcription regulator AtxA, which controls anthrax toxins and PDGA capsule in *B. anthracis*. Thus, the HA and PDGA capsules and toxins were co-regulated by AtxA. We explored the respective effect of the virulence factors on colonisation and dissemination of CA within its host by constructing bioluminescent mutants. Expression of the HA capsule by itself led to local multiplication and, during intranasal infection, to local dissemination to the adjacent brain tissue. Co-expression of either toxins or PDGA capsule with HA capsule enabled systemic dissemination, thus providing a clear evolutionary advantage. Protection against infection by *B. cereus* bv *anthracis* required the same vaccination formulation as that used against *B. anthracis*. Thus, these strains, at the frontier between *B. anthracis* and *B. cereus*, provide insight into how the monomorphic *B. anthracis* may have emerged.

## Author Summary

Anthrax is caused by the bacterium *Bacillus anthracis* that affects all mammals worldwide. It emerged more than 10,000 years ago from a *Bacillus cereus* precursor. In the past decade, *B*. *cereus* bacteria were isolated in the USA from anthrax-like pneumonia cases. They harbour one virulence plasmid very similar to the toxin–encoding plasmid of *B*. *anthracis*. Recently, an anthrax-like disease in great apes in Africa was caused by emerging *B*. *cereus* strains, named *B*. *cereus* biovar *anthracis*. These strains are atypical as they possess both plasmids coding for toxin and capsule similar to those so far found only in *B*. *anthracis*. These unusual pathogenic *B*. *cereus* are currently neglected. We explored the virulence of these pathogens and their colonisation and dissemination capacity within the murine host. We found that these toxinogenic strains harbour two capsules, the classical *B*. *anthracis* capsule and an additional polysaccharidic capsule. This latter capsule confers virulence alone or in combination with toxins. Both capsules are concomitantly expressed, under the control of a common global regulator and host signals. Our results show that acquisition of new genetic information by these *B*. *cereus* clearly gives them a selective advantage, favouring their dissemination within infected hosts and the environment.

## Introduction

*Bacillus cereus* and *Bacillus anthracis* are genetically closely related and belong to the *B*. *cereus* group [1,2]. *B*. *cereus* strains display a dynamic genome and carry numerous mobile genetic elements, including several plasmids [2]. Genetic exchange is frequent between bacteria of the *B*. *cereus* lineage [2,3,4,5]. Most *B*. *cereus* isolates are non pathogenic. Some strains however may cause food poisoning, due to production of toxins, emetic toxin or enterotoxins; *B*. *cereus* can also cause opportunistic and nosocomial infections especially in immunocompromised individuals [2]. *B*. *anthracis*, the etiological agent of anthrax, is highly monomorphic and possesses specific pathogenic properties that differentiate it from *B*. *cereus* [2]. The *B*. *anthracis* lineage is genetically defined by particular chromosomal-borne characteristics. These include four chromosomal lambdoid prophages and a particular nonsense mutation in the *plcR* gene resulting in the absence of a functional PlcR protein, which is a global regulator in the *B*. *cereus* group [2,6].

Anthrax is an acute lethal disease associating infection and toxemia [7]. The virulence properties of *B*. *anthracis* that are characteristic of anthrax are due to the presence of two particular plasmids, pXO1 and pXO2, which are responsible for toxin production and capsule synthesis, respectively. The *pagA*, *lef* and *cya* genes, located within a 44.8 kb pathogenicity island (PAI) [8] on pXO1, code for the tripartite toxin components: the protective antigen PA, the lethal factor LF, and the edema factor EF. These proteins act in combination to form the lethal toxin (LT) and the edema toxin (ET) [9]. The pXO2 plasmid carries the *cap BCADE* operon, encoding the enzymes involved in the synthesis and anchoring of the poly-γ-D-glutamate capsule (PDGA). This capsule inhibits phagocytosis, functions as a non-immunogenic surface, enables bacterial adhesion to the endothelium and is essential for virulence [9,10,11]. AtxA is a global regulator of virulence that is encoded by *atxA* located within the PAI on pXO1. AtxA activates the transcription of toxin and capsule genes, at 37°C in the presence of CO_2_ and bicarbonate [12].

It is generally considered that *B*. *anthracis* strains differ from other strains in the *B*. *cereus* group by the presence of pXO1 and pXO2. However, the emergence of *B*. *cereus* strains that possess pathogenic properties similar to those of *B*. *anthracis* has blurred the frontiers between the two species [13,14]. At least four *B*. *cereus* isolates (G9241, 03BB87, 03BB102 and Elc2) have been associated with severe to fatal pneumonia resembling cases of inhalational anthrax [13,14,15,16]. They were isolated from metal workers living in different parts of Texas and Louisiana, over a period of more than a decade. These strains harbour a pXO1-like plasmid, named pBCXO1, that shows 99.6% similarity to pXO1 and therefore expresses the anthrax toxin genes. They harbour a functional PlcR [13,16] and express various genes belonging to the PlcR regulon, in particular those encoding haemolysins, proteases and phospholipases [2,6,17]. Although they are encapsulated, their capsule is not made of poly-γ-D-glutamate [15], but is instead of a polysaccharide nature [13]. The G9241 strain carries a second large plasmid, pBC218, which carries the *bpsX-H* operon encoding a polysaccharide capsule [13,18]. This plasmid, or one very similar to it, has been found in the 03BB87 strain [13]. Furthermore, an operon, *hasACB*, located on pBCXO1 and encoding a hyaluronic acid capsule, is functional in the G9241 strain, in contrast to *B*. *anthracis* in which the *hasA* gene is mutated [18,19]. The G9241 strain is therefore able to synthesize two polysaccharide capsules [18].

Emerging *B*. *cereus* strains have been isolated from great apes that died from an anthrax-like disease in Cameroon (CA) and Côte d’Ivoire (CI) [20,21,22,23]. These unusual strains are unlike any other isolated before and were originally designated as “*Bacillus cereus* variety *anthracis*”, but were later renamed *B*. *cereus* biovar (bv) *anthracis* to comply with database requirements. Their genomic characterization showed that they belong to the *B*. *cereus* species although they harbour two plasmids, pBCXO1 and pBCXO2 (initially designated as pCI-XO1 and pCI-XO2, respectively), that are highly similar to the pXO1 and pXO2 *B*. *anthracis* plasmids [20,23,24]. These strains produce active edema and lethal toxins and they are encapsulated with a PDGA capsule [20,24]. These features clearly indicate that plasmids no longer unambiguously differentiate *B*. *anthracis* from other members of the *B*. *cereus* group. The CA and CI strains do not carry the *plcR* mutation specific to *B*. *anthracis*. However, these strains do contain a frameshift mutation at the 3’ end of the gene that creates a four amino-acid extension at the C-terminus of the protein [24]. The mutant protein is probably inactive, as these strains do not express the degradative enzymes controlled by PlcR and are non-haemolytic. Therefore, the CA and CI strains possess phenotypic properties characteristic of *B*. *anthracis* which makes these strains difficult to classify [23,24].

Most recently, anthrax was considered among the three major neglected zoonotic diseases that need recognition [25]. This is even truer to the atypical pathogenic *B*. *cereus* CA and CI strains whose virulence and interactions with the host remain to be elucidated. In this study, we characterise the virulence factors implicated in the pathogenicity of the CA and CI strains and describe their effect on colonisation and dissemination within the host. We show that the CA and CI strains harbour two concomitantly expressed capsules, a PDGA capsule similar to that of *B*. *anthracis*, and a hyaluronic acid (HA) capsule. The genes involved in the production of both capsules are regulated by the global regulator AtxA. We explored the role of each capsule and toxin, as well as their concerted action *in vivo*.

## Materials and Methods

### Ethics statement

All the animal experiments described in the present study were conducted at the Institut Pasteur according to the European Union guidelines for the handling of laboratory animals (http://ec.europa.eu/environment/chemicals/lab_animals/home_en.html) and were approved by the Institut Pasteur animal care and use committee (CETEA n°2013–0088). All efforts were made to minimize suffering.

### Bacterial strains and growth conditions

*B*. *cereus* bv *anthracis and Bacillus anthracis* strains used in this study are listed in Table 1. *Bacteria* were grown in Brain Heart Infusion (BHI) broth or on BHI agar (Difco, http://www.bd.com/ds) unless otherwise noted. Antibiotics were used when necessary at the following concentrations: spectinomycin at 100 μg/ml and erythromycin at 5 μg/ml. *B*. *cereus* bv *anthracis* and *B*. *anthracis* spores were produced as described previously [26].

**Table 1:** *B. cereus* bv *anthracis* and *B. anthracis* strains used in this study.

**Strains**	**Genotype or description**	**Source or reference**
CI	pBCXO1^+^; pBCXO2^+^; pCI-14	Klee *et al*., 2006
CA	pBCXO1^+^; pBCXO2^+^	Klee *et al*., 2006
CAP	pBCXO1^+^, ΔpagA; pBCXO2^+^; Spc^r^	This study
CA-H	pBCXO1^+^, ΔhasA; pBCXO2^+^; Spc^r^	This study
CAR	pBCXO1^+^	This study
CAR-P	pBCXO1^+^, ΔpagA; Spc^r^	This study
CAR-R	pBCXO1 & pBCXO2 cured	This study
CAR-H	pBCXO1^+^, ΔhasA; Spc^r^	This study
CAR-20	pBCXO1^+^, atxA*	This study
CAR-20*atxA*	pBCXO1^+^, pDACatxA::Pspac-atxA; Spc^r^	This study
CAP*lux*	pBCXO1^+^, ΔpagA, PpagA-lux; pBCXO2^+^; Spc^r^, Erm^r^	This study
CAR*lux*	pBCXO1^+^, PpagA-lux; Erm^r^	This study
CAR-P*lux*	pBCXO1^+^, ΔpagA, PpagA-lux; Spc^r^, Erm^r^	This study
*Ba*7702	Sterne strain, 34F2; pXO1^+^	Laboratory stock
*Ba*9602	pXO1^+^; pXO2^+^	Berthier *et al*., 1996
*Ba*Vollum	pXO1^+^; pXO2^+^	ATCC 14578**

atxA*: Inactive *atxA* through spontaneous mutations; *Ba*: *B*. *anthracis*

**Kindly provided by Wolfgang Beyer, Hohenheim University, Stuttgart, Germany.

### Construction of *B*. *cereus* bv *anthracis* CA mutant strains

The presence of the plasmids pBCXO1 and pBCXO2 was verified by multiplex PCR analysis [27]. The CA strain was cured of the pBCXO2 plasmid as described for *B*. *anthracis* [28], resulting in the CAR strain. Loss of pBCXO2 was confirmed by multiplex PCR. Mutant strains were constructed by heterogramic conjugation and the conjugative *E*.*coli* strain HB101 (pRK212.1) was used to transfer the relevant plasmids [29], as described previously for the construction of *B anthracis* mutant strains [30,31,32]. CAP and CAR-P mutants in which the *pagA* gene is inactive were constructed as described for the 9602P *B anthracis* strain [33]. The bioluminescent derivatives CAR-P*lux*, CAR*lux* and CAP*lux* were constructed through conjugation with involving the pIG6–19 plasmid carrying the *luxABCDE* operon of *Photorhabdus luminescens* under the control of the *pagA* promoter, as described for the construction of the *B*. *anthracis* bioluminescent strains [30]. The replicative pDAC-*atxA* plasmid [34], which contains *atxA* under the control of the *Pspac* promoter [35] was used for AtxA complementation experiments.

For the construction of CA-H(Δ*hasA*) and CAR-H(Δ*hasA*) mutant strains, the *hasA* gene sequences from position 106 to 712 and from position 2012 to 2527 were amplified from CAR genomic DNA with the following primers: GGTACCCTAATAATTATCTATGGAAGTGGAGGAGGG / CCCGGGCGATCTAAATT TCTCATAGGACGTATAAGG for fragment 106–712 (KpnI and XmaI sites underlined) and CCCGGGGCTTTACTAACTATTAAATCTAATGGTTGG / CTGCAGCAATTGCTTGA GACATGGATACGTAGAGCT for fragment 2012–2527 (XmaI and PstI sites underlined). The resulting PCR products were inserted into the pGEM-Teasy plasmid which was introduced into TG1 *E*. *coli*. These *hasA* gene sequences were digested with KpnI/XmaI or XmaI/PstI and were inserted sequentially into the conjugative suicide plasmid pAT113 cut by the same enzymes [36], resulting in the pCB12 plasmid. The pUC1318 spectinomycin resistance cassette was extracted through SmaI/HincII digestion and was inserted into the SmaI-digested pCB12 plasmid. The resulting plasmid pCB12sp was transferred through conjugation into the CA and CAR strains. The inactivation of the *has*A gene in the resulting recombinant strains was confirmed by PCR with appropriate primers and phenotypic characterisation (Alcian blue and India ink staining).

### RNA isolation and expression analysis

Bacterial precultures were grown overnight in BHI broth (37°C, 200 rpm). Experimental cultures were diluted 1:25 in fresh medium and subcultivated either in BHI broth in ambient air or in BHI broth containing 0.8% (w/v) sodium bicarbonate in 5% CO_2_ atmosphere. *AtxA* expression was induced in the pDAC-*atxA* complemented strain (Table 1) by adding IPTG (1 mM final concentration) one hour after subcultivation.

The bacterial pellet corresponding to 1 ml of bacterial culture in late log phase was resuspended immediately in boiling lysis buffer (2% SDS, 16 mM EDTA, 20 mM NaCl) and incubated for 5 min at 95°C. After addition of 3 M sodium acetate on ice and cooling, nucleic acid extraction was performed with a 25:24:1 mix of phenol:chloroform:isoamyl alcohol. Samples were incubated for 15 min on ice and were centrifuged (12000 x g at 4°C for 5 min). The upper phases were recovered and a 1/10 volume of each 3 M sodium acetate and 1 mM EDTA and 2.5 volumes of ice-cold ethanol were added. Samples were incubated at -80°C for at least one hour to precipitate RNA. After centrifugation (12000 x g at 4°C for 30 min), pellets were resuspended in RNase-free water and RNA was further purified with an RNeasy Mini Kit (Qiagen, Hilden, Germany) according to the manufacturer´s instructions. After elution with RNase-free water, total RNA concentrations were measured by UV spectrophotometry (Nanodrop, Thermo Scientific, Wilmington, USA) and RNA quality was assessed by measuring the ratio of absorbance at 260 and 280 nm.

For reverse transcriptase (RT)-PCR, purified RNA (3 μg) was treated with DNase according to manufacturer's protocol (Fermentas, St. Leon-Roth, Germany) and the absence of contaminating DNA was verified by PCR. Reverse transcription was carried out with 1 μg of DNA-free total RNA for 1 h at 42°C. The M-MLV RT-System (Invitrogen) was used and the reaction additionally contained 2 μl (10 μM) gene specific cDNA-primer (Table 2, cDNA), 2 μl (10 mM) dNTPs and 1 μl RNase inhibitor (Fermentas, St. Leon-Roth, Germany).

**Table 2:** Oligonucleotides for expression analysis used in this study.

**Oligonucleotides**	**Sequence**
hasA- cDNA	GTACGATTAAGTCAGGAATACC
hasA-for	TAAACCTTATACGTCCTATGAG
hasA-rev	TGAGTTCTAAGAAGTTCCTCC
capB-cDNA	GATAATCGGGTTGAACTGCC
capB-for	GGGAAAACAACTGGTACATCTGC
capB-rev	AAGTGCTTCTGCTTCTAAATCAGC
gyrB-cDNA	TCATGTGTTCCACCTTCATAC
gyrB-for	CAGGGTACTGTGACGAAATTAACG
gyrB-rev	CACCATGCAAACCACCAGAAAC

PCR amplification of the *hasA*, *capB* and *gyrB* gene fragments was performed in a 25 μl final volume with 2.5 μl 10X buffer, 0.2 mM of each dNTP, 1.5 mM MgCl_2_, 0.2 μM concentrations of each primer (Table 2, for and rev), 0.6 units of Taq polymerase (Fermentas, St. Leon-Roth, Germany), and 1 μl of cDNA. The PCR program consisted of one step at 94°C for 5 min, followed by 35 cycles consisting of 94°C for 30 s, 55°C for 30 s and 72°C for 30 s, and a final step at 72°C for 10 min. The PCR products were separated on a 1.5% agarose gel and were stained with ethidium bromide. The size of the PCR product of *hasA*, *capB* and *gyrB* were 456 bp, 144 bp and 195 bp, respectively. All primers were supplied by Metabion (Martinsried, Germany).

### Sequence analysis

For sequencing of the *has* operon and the *atxA* gene, PCR fragments were generated from genomic DNA of the CAR, CAR-P and derivative strains (S1 Table), were processed by standard procedures [37] and were then sequenced with an AB3100 DNA sequencer (Applied Biosystems, Foster City, USA). Sequences were compiled and analysed with the Gap4 software (The Staden package, 1998—PMID: 10547834).

### Characterisation of capsules and toxins

Capsule-inducing agar plates were used as described previously [28,38]. The presence of a capsule was visualised under a microscope after India ink staining. Where required, bacterial cultures were incubated with 200 units of hyaluronidase (H3506, Sigma-Aldrich, Lyon, France), for 30 min in sodium phosphate buffer before India ink staining. The PDGA capsule was detected by immunofluorescence labelling of bacteria with a PDGA-specific polyclonal antiserum as previously described [10].

Bacterial cultures were grown overnight at 37°C on BHI agar containing 0.8% (w/v) sodium bicarbonate in a 5% CO_2_ atmosphere. IPTG (1 mM final concentration) was added to the agar to induce *atxA* gene expression in the CAR20-pDAC-*atxA* strain. One loop of colony material was resuspended in 0.5 ml phosphate-buffered saline (PBS, pH 7.2). Where required, 50 μl of a hyaluronidase (Sigma-Aldrich, Taufkirchen, Germany) stock solution (10 mg/ml in PBS, corresponding to 4,000–10,000 Units per ml according to the manufacturer) was added to the bacterial suspension and incubated for 30 min at 37°C. The reaction was stopped by heating at 95°C for 10 min. The colony suspension was centrifuged for 20 min at 12000 x g and the supernatant containing capsule material was filter-sterilised (0.2 μm pore size). The samples were analysed by 5% sodium dodecyl sulfate (SDS) polyacrylamide gel electrophoresis (PAGE) by standard procedures (running buffer: 25 mM Tris, 192 mM glycine, 0.1% SDS, pH 8.3). Prior to loading, 7.5 μl of the supernatant was mixed with 2.5 μl of 4 x Laemmli loading buffer and was heated at 95°C for 5 min. After electrophoresis at 20 mA for approximately 2 hours, the gel was fixed in 10% acetic acid, 40% ethanol for at least 1 hour. The fixing solution was replaced by Alcian blue (Sigma-Aldrich) solution (0.025% Alcian blue in fixing solution) and gently agitated for 30 min. The gel was then destained in fixing solution [39,40]. The stained gels were visualised with the ChemiDoc MP System with Image Lab Software (BioRad, Munich, Germany).

Toxin components PA and LF were detected by western blot of bacterial culture supernatants derived from cultures grown overnight in R medium supplemented with 0.8% bicarbonate in 5% CO_2_ at 37°C. The monoclonal antibodies against PA and LF and the western blotting procedure were described previously [33].

### Determination of virulence and immunoprotection

Outbred OF1 female mice (6 to 8 weeks old) and Hartley guinea pigs (200 to 250 g) were purchased from Charles River (L'Arbresle, France). The animals were housed in the BSL3 animal facilities of the Institut Pasteur licensed by the French Ministry of Agriculture and complying with European Union regulations. The protocols were approved by the Institut Pasteur Safety Committee, according to the standard procedures recommended by the Institut Pasteur Animal Care and Use Committee. Mice and guinea pigs were infected subcutaneously in the flank with graded doses of spores in 200 μl PBS. Intranasal inoculation was performed in lightly anesthetised animals by depositing onto the nostril graded doses of spores in 20 μl of PBS. CFU number in the inoculum was retrospectively checked by plating 10-fold dilutions on BHI agar. Survival was monitored twice daily for at least 15 days. All calculations of virulence were performed at least twice. Mean lethal doses (LD_50_) were calculated by the method of Reed and Muench [41].

Immunoprotection experiments on mice were performed as previously described [42,43]. Briefly, 200 μl of a mix of 10 μg of recombinant PA (a kind gift from Dr Bassam Hallis, Public Health England, Porton Down, UK) and 1 x 10^8^ formaldehyde inactivated *B*. *anthracis* spores of the RPLC2 strain (FIS), with 0.3% aluminium hydroxide gel (Sigma-Aldrich, St. Louis, MO) in PBS were injected subcutaneously into the flank at day 0 and 14. Challenge with spores of the *B*. *cereus* bv *anthracis* or *B*. *anthracis* strain was performed subcutaneously at day 35 and survival was monitored for 20 days.

### Bioluminescence imaging

Cutaneous infection was performed by injecting 10 μl of spore suspension in PBS into the dermis of the right ear pinna as described previously, or by depositing 20 μl of spore suspension onto the nostril [30]. Images were acquired with an IVIS 100 system (Xenogen) according to instructions from the manufacturer. Analysis and acquisition were performed with Living Image 2.5 software (Xenogen). Unless otherwise noted, mice were anaesthetised with a constant flow of 2.5% isofluorane mixed with oxygen. The XGI-8 Gas Anaesthesia System (Xenogen) was used, which enabled control of the duration of anaesthesia. Images were acquired with a binning of 16. Luminescent signals from the exterior of mice were acquired for 1 min. All other photographic parameters were kept constant.

### Electron microscopy

Encapsulated bacterial cells were fixed with 2.5% glutaraldehyde in 0.1 M cacodylate—5 mM CaCl_2_ buffer (pH 7.2) as described previously and were postfixed for 2 h with 2% OsO_4_ in the same fixation buffer [44]. The pelleted bacteria were embedded in 2% low-melting-point agar (type IX; Sigma). The samples were then treated with 0.5% uranyl acetate in water for 1 h. After extensive washing, small blocks were dehydrated with alcohol and embedded in Epon. Thin sections were conventionally poststained and observed with a Jeol 1010 electron microscope.

### Statistical analysis

Data were processed with GraphPad PRISM 4 software to generate graphs and to carry out statistical analyses. Statistical evaluation of survival was performed with the log-rank test. Results were expressed as mean values ± SD. Statistical significance was determined by Student’s *t* test.

## Results

### Contribution of PDGA capsule and toxins to the virulence of *B*. *cereus* bv *anthracis*

*B*. *cereus* bv *anthracis* CA and CI strains harbour two virulence plasmids, pBCXO1 and pBCXO2, that are highly similar to the virulence plasmids pXO1 and pXO2 characteristic of *B*. *anthracis*. We thus examined the virulence of these two strains in two complementary animal models, mice and guinea pigs, and by the two main routes of infection, subcutaneous and intranasal.

When the CA and CI strains were delivered by the subcutaneous route, the LD_50_ of the two strains was similar both in mice (60 spores for CA and 130 spores for CI; Table 3) and in guinea pigs (100 spores for CA and 300 spores for CI; Table 4). These values are similar to those reported for the fully virulent *B*. *anthracis* 9602 strain (<25–40 spores in mice and 65 spores in guinea pigs [45]). When the CA and CI strains were delivered by the intranasal route in mice, both strains showed similar virulence with an LD_50_ close to the value for the *B*. *anthracis* 9602 strain (Table 3). We examined only the CA strain by the intranasal route in the guinea pig. The LD_50_ was similar to that of the 9602 strain (1.2 x 10^7^ spores for CA vs 2 x 10^7^ spores for strain 9602) with a mean time to death of 5.9 ± 2.1 days (n = 6) for CA vs 4.0 ± 0.3 days (n = 6) for strain 9602.

**Table 3:** Virulence of the *Bacillus cereus* bv *anthracis* and the CA derivative strains in mice.

**Route**	**Strain**	**pBCXO1**	**pBCXO2**	**Virulence factors**	**LD50**	**MTD (days)**
Cutaneous	CI	+	+	Tox, HA, PDGA	130	3.2 ± 0.4
	CA	+	+	Tox, HA, PDGA	60	4.4 ±1.8
	CA-P	Δ*pagA*	+	HA, PDGA	70	4.7 ± 1.2
	CAR	+	-	Tox, HA	550	3.4 ± 0.4
	CA-H	Δ*hasA*	+	Tox, PDGA	70	3.4 ± 0.5
	CAR-P	Δ*pagA*	-	HA	6 x 10^7^	2.1 ± 0.7
	CAR-H	Δ*hasA*	-	Tox	3 x 10^6^	5.0 ± 1.3
	*Ba*7702	pXO1	-	Tox	4 x 10^5^	3.75 ± 0.9
	CAR-R	-	-	-	> 6 x 10^7*^	NA
	CAR20	+	-	-	> 1 x 10^8*^	NA
Intranasal	CI	+	+	Tox, HA, PDGA	3.5 x 10^4^	3.1 ± 1.6
	CA	+	+	Tox, HA, PDGA	3.5 x 10^4^	4.2 ± 0.7
	CAR	+	-	Tox, HA	2.5 x 10^4^	4.5 ± 0.7
	CA-H	Δ*hasA*	+	Tox, PDGA	2.2 x 10^4^	3.6 ± 1.8
	CAR-P	Δ*pagA*	-	HA	1 x 10^7^	4.1 ± 1.5
	CAR-H	Δ*hasA*	-	Tox	> 1 x 10^8*^	NA
	*Ba*7702	pXO1	-	Tox	> 1 x 10^8*^	NA
	*Ba*9602	pXO1	pXO2	Tox, PDGA	1 x 10^4^	2.8 ± 0.7

Mice were inoculated with graded spore inocula of each strain in the flank or by the intranasal route (six animals per dose). The following information is specified where applicable: (i) the presence of pBCXO1, PBCXO2 for the CI and CA-derived strains, and pXO1, pXO2 for the *B*. *anthracis (Ba)* Sterne 7702 and wild-type 9602 strains; (ii) the gene inactivated on pBCXO1; and iv) the virulence factors expressed—lethal and edema toxins (Tox), hyaluronic acid capsule (HA) and polyglutamic acid capsule (PDGA)—is indicated for each mutant. Results are expressed as mean lethal dose (LD50) and mean time to death in days (MTD, mean ± SD). Each experiment was performed at least twice. The asterisk denotes absence of mortality at the highest inoculum tested. NA, not applicable.

**Table 4:** Virulence of the *Bacillus cereus* bv *anthracis* and CA derivative strains by the subcutaneous route in guinea pigs.

**Strain**	**pBCXO1**	**pBCXO2**	**LD50**	**MTD (days)**
CI	+	+	300	5
CA	+	+	100	3.1 ± 0.3
CAP	Δ*pagA*	+	1 x 10^4^	4.75 ± 1
CAR	+	-	2 x 10^3^	5.2 ± 1.2

Guinea pigs were inoculated with graded spore inocula of each strain by subcutaneous route in the flank (four animals per dose). The presence of pBCXO1 and PBCXO2 and the gene inactivated on pBCXO1 is specified where applicable. Results are expressed as mean lethal dose (LD50) and mean time to death in days (MTD, mean ± SD). Each experiment was performed at least twice.

**Fig 1:**
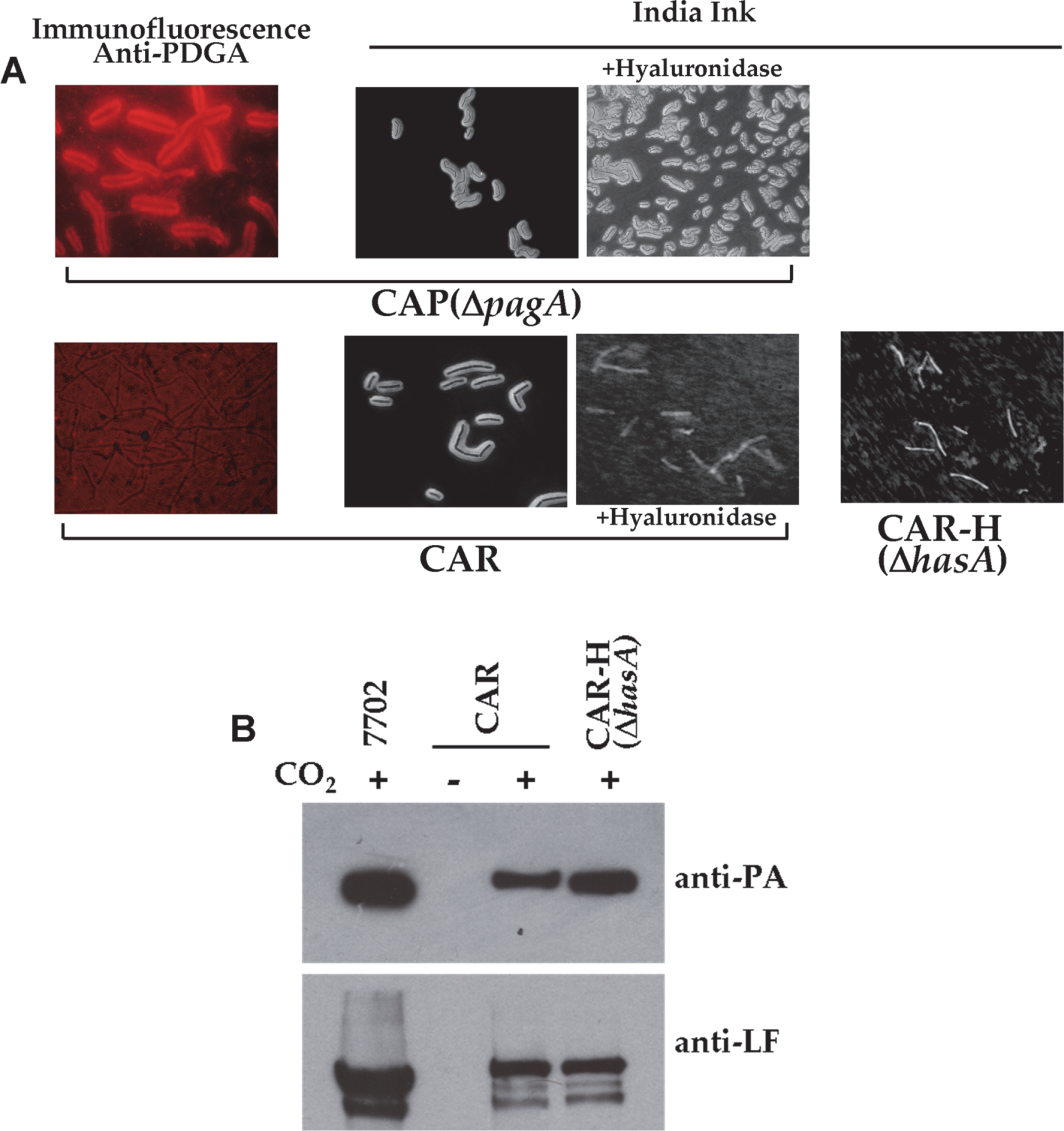
The *B*. *cereus* bv *anthracis* CA strain expresses a PDGA and a HA capsule, and toxins. **(A)** Capsule expression in the CAP(Δ*pagA*), the CAR and the CAR-H(Δ*hasA*) strains in inducing conditions; the polyglutamate (PDGA) and hyaluronic acid (HA) capsule was visualised by immunofluorescence with a polyclonal anti-PDGA immune serum or by India ink staining; degradation of the HA capsule was achieved by incubation with hyaluronidase as described in the Materials and Methods section. (**B)** The production of toxin components PA and LF in overnight bacterial culture supernatants was determined by western blot with or without CO_2_/bicarbonate as described in the Materials and Methods section.

We then investigated the respective contribution of each main virulence factor, the polyglutamate (PDGA) capsule and the toxins, to the virulence of the *B*. *cereus* bv *anthracis* CA strain. The virulence of a CAP(Δ*pagA*) mutant lacking protective antigen and therefore toxin activity (CAP strain, Table 3) was not modified in mice (LD_50_ by subcutaneous route 70 spores vs 60 spores for the parental CA strain) and its virulence was a 100-fold attenuated in the guinea pig (10^4^ for the mutant vs 10^2^ for the CA strain; Table 4). To analyse the contribution of the polyglutamate capsule, the CA strain was cured of the pBCXO2 plasmid, resulting in the CAR strain. The absence of the PDGA capsule was confirmed by the absence of immunofluorescence following the labelling of bacteria with a PDGA-specific polyclonal antiserum (Fig. 1A, [10]). Toxin production in inducing conditions was not modified, as shown by immunoblotting for PA and LF (Fig. 1B). Most strikingly, loss of the pBCXO2 plasmid did not modify virulence when bacteria were delivered intranasally in mice, and the CAR strain remained as virulent as the parental CA strain (Table 3). Furthermore, the virulence of CAR was only slightly attenuated in mice and guinea pigs inoculated by the subcutaneous route (LD_50_ of CAR was 8-fold higher than that of CA in mice and was 10-fold higher in guinea pigs; Tables 3 and 4). These results were unexpected, because curing *B*. *anthracis* of pXO2 (as in the 7702 Sterne strain) results in the absence of virulence when the Sterne strain is delivered by the intranasal route and a significant attenuation of virulence by the subcutaneous route (Table 3 with a LD_50_ for the 7702 Sterne strain of 4 x 10^5^; [46,47]. These observations led to the development of the veterinary Sterne vaccine strain after 1939 [48]. These properties of the CAR strain therefore suggest the presence of additional virulence factor(s) in the parental CA strain.

### Identification of a pBCXO1-encoded hyaluronic acid capsule as an additional virulence factor for CA

CAR colonies grown on capsule-inducing agar medium still showed a somewhat smooth appearance, although this was less apparent than with the parental CA strain. We confirmed the presence of capsular material surrounding the CAR bacteria by India ink staining (Fig. 1A). We isolated a spontaneous rough colony of the CAR strain (CAR-R strain) on capsule-inducing agar plates; the lack of any capsule was confirmed by India ink staining of CAR-R bacteria. Multiplex PCR analysis of this rough clone showed that pBCXO1 was lost, suggesting that the expression of the capsule in the CAR strain was linked to the presence of pBCXO1 (S1A Fig.).

We then investigated whether this capsule may be the pBCXO1-encoded hyaluronic acid capsule (*has* operon), as recently reported for the pathogenic *B*. *cereus* G9241 strain [18]. DNA sequencing of the *hasACB* operon of the CAR strain showed that, in contrast to *B*. *anthracis* [8], the *hasA* gene is non-mutated and the deduced sequence of the HasA protein is identical to the sequences available in the databases for the CI and G9241 strains. The HasC and HasB protein sequences were also identical to those of the CI and G9241 strains, with only one mismatch for HasB at position 209 (Ala→Thr) compared with the sequence from G9241.

We examined the role of the *hasACB* operon in the capsule produced by the CAR strain by inactivating the *hasA* gene encoding the hyaluronate synthase. The resulting CAR-H(Δ*hasA*) strain gave rough colonies on capsule-inducing agar and showed no capsule by India ink staining, suggesting that it was unable to synthesise a capsule (Fig. 1A). Hyaluronidase enzymatic digestion of encapsulated bacteria confirmed the hyaluronic acid nature of this *has* operon-encoded capsule in the CAR strain; this treatment led to a complete absence of the capsule as visualised in Fig. 1A. The CA strain, and the CI (through database sequence analysis [24]), thus harbour genetic determinants encoding a PDGA capsule on pBCXO2 and a HA capsule on pBCXO1.

**Fig 2:**
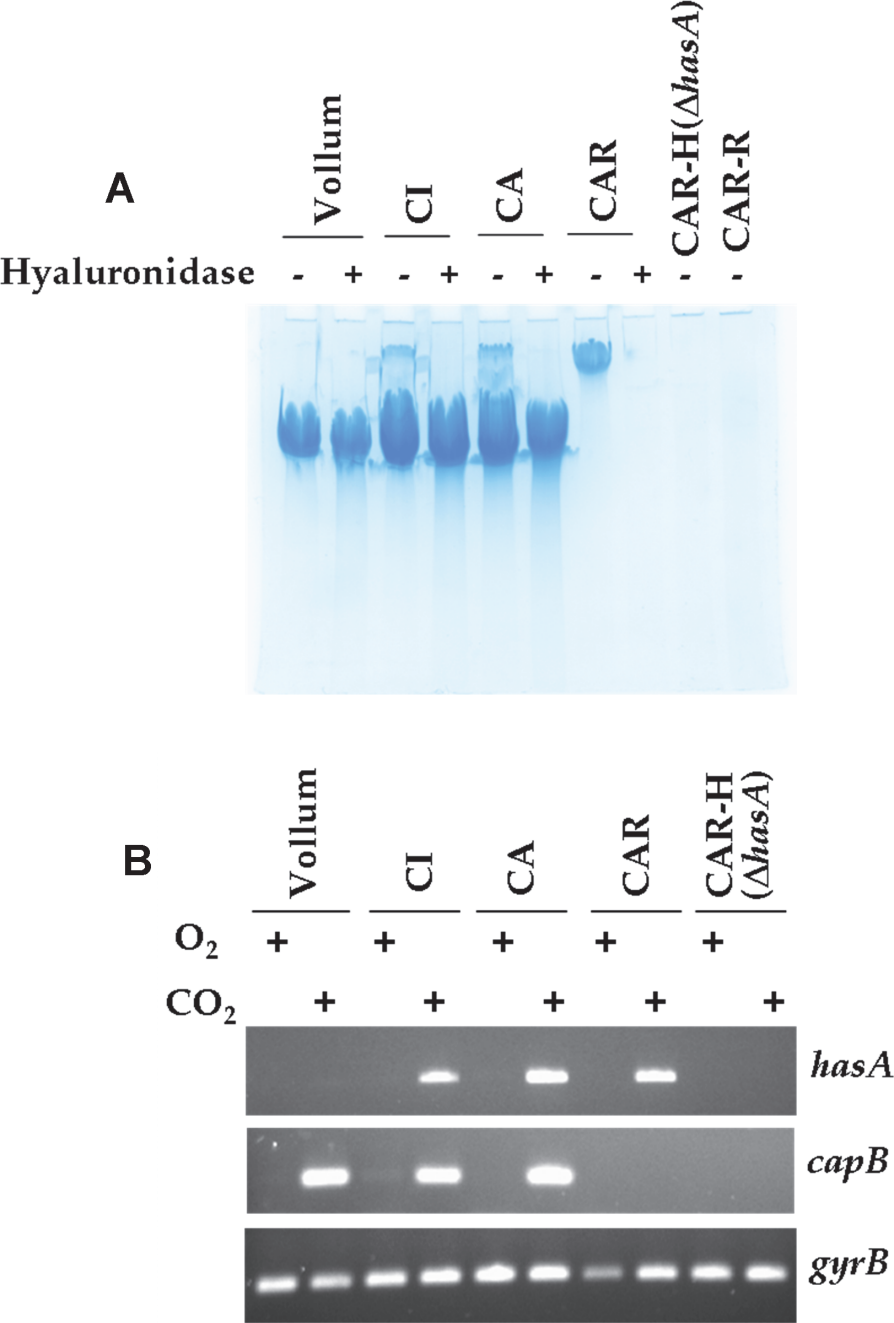
Coexpression of a PDGA and a HA capsule by the *B*. *cereus* bv *anthracis* strains. **(A)** Alcian Blue staining was performed on filtrates of colony lysates from various strains grown in CO_2_/bicarbonate conditions: these were the Vollum strain (wild-type *B*. *anthracis)*, the *B*. *cereus* bv *anthracis* CI and CA strains, and the CA-derived strains devoid of pBCXO2 (CAR) and further deleted in the *hasA* gene (CAR-H) or having lost pBCXO1 (CAR-R); hyaluronidase treatment was performed before PAGE, as described in the Materials and Methods section. **(B)** mRNA of the *hasA* gene (involved in synthesis of the HA capsule) and the *capB* gene (involved in synthesis of the PDGA capsule) was assessed in the strains described in (A) grown under CO_2_/bicarbonate (CO_2_) or aerobic (O_2_) culture conditions as described in the Materials and Methods section; *gyrB* gene expression was used as reference.

We visualised the capsules produced by the CA and CI strains through Alcian blue staining. SDS-PAGE with low concentrations of acrylamide revealed two high molecular weight bands in bacterial extracts of these strains (Fig. 2A). The lower band was present in all PDGA-expressing strains, i.e. the PDGA-encapsulated *B*. *anthracis* Vollum strain and the CA and CI strains; it was absent in the strains that do not produce a PDGA capsule, i.e. the pBCXO1+ pBCXO2-cured CAR strain and the plasmid-free CAR-R strain. The upper band was present in the CA and CI strains, and in the CAR strain that expresses a HA capsule; it was absent from the CAR-H(Δ*hasA*) and CAR-R strains, and from the *B*. *anthracis* Vollum strain, that do not produce the HA capsule. Furthermore, hyaluronidase enzymatic digestion of the bacterial extracts led to the disappearance of the upper band for the CI, CA and CAR strains (Fig. 2A).

These data suggest that the two high molecular weight bands detected in the CA and CI samples represent the PDGA (lower band) and the HA (upper band) capsular material. Furthermore, this shows that, in inducing conditions, both capsules are concomitantly expressed at the CA and CI bacterial surface. To address the relative contribution of each capsule to virulence in the presence of toxins, the *hasA* gene was inactivated in the CA strain, leading to a PDGA-encapsulated mutant; its virulence was not modified both by subcutaneous and intranasal routes in mice (Table 3, CA-HΔ*hasA* strain). As shown above (Table 3), the virulence of the HA-encapsulated CAR strain was unaffected by intranasal route, though a slight reduction was observed by subcutaneous route. Thus each capsule contributes to virulence in the presence of toxins and may favour entry through inhalational route.

**Fig 3:**
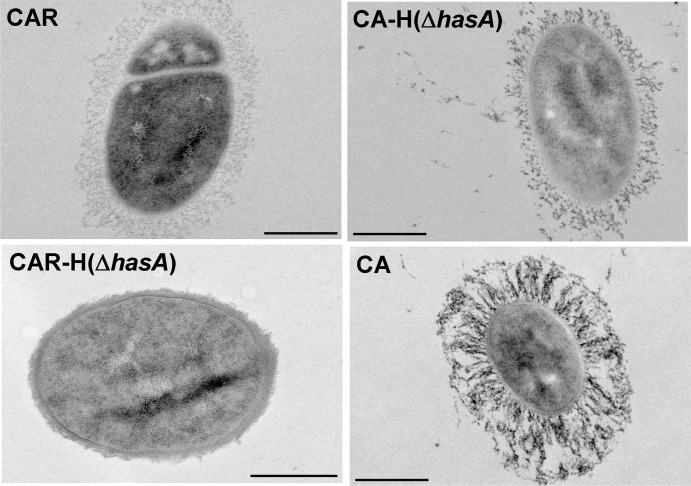
Ultrastructural analysis of the capsules of the *B*. *cereus* bv *anthracis* CA strain and its derivatives. Bacterial cells from the PDGA and HA capsule-expressing CA strain and its derivatives expressing a HA capsule (CAR), a PDGA capsule (CA-H(Δ*hasA*)) or no capsule (CAR-H(Δ*hasA*)) were prepared for Transmission Electron Microscopy as described in the Materials and Methods section. Scale bar: 500nm

Ultrastructural analysis by TEM (Fig. 3) showed the HA-capsule consists of fine filaments of homogenous density surrounding the entire bacterial CAR surface. This structure was absent in the CAR-H(Δ*hasA*) mutant (Fig. 3). In a CA-H(Δ*hasA*) mutant, which lacks the HA-capsule but produces a PDGA-capsule, the organisation of the filaments surrounding the bacteria was distinct from that observed for CAR (Fig. 3). In the parental CA strain, which produces the two types of capsules, the resulting capsular structure appeared different from the structures surrounding the HA-expressing CAR and PDGA-expressing CA-HΔ*hasA* strains (Fig. 3).

### Co-regulation of the expression of the HA and PDGA capsules

We only observed the encapsulated phenotype of the CAR strain when CO_2_/Bicarbonate was present in the culture conditions, either in solid or liquid media. This suggests that inducing conditions similar to those described for the PDGA-capsule in *B*. *anthracis* are required for the expression of the HA-capsule. Molecular analysis by RT-PCR showed that the *hasA* transcript in the CA, CI and CAR strain was present only when cells were cultured under CO_2_/Bicarbonate inducing conditions, and was absent, as expected, in the CAR-H(Δ*hasA*) strain (Fig. 2B). In the CA and CI strains, both *hasA* and *capB* transcripts were detected under the same inducing conditions (Fig. 2B), indicating that their expression is coregulated.

**Fig 4:**
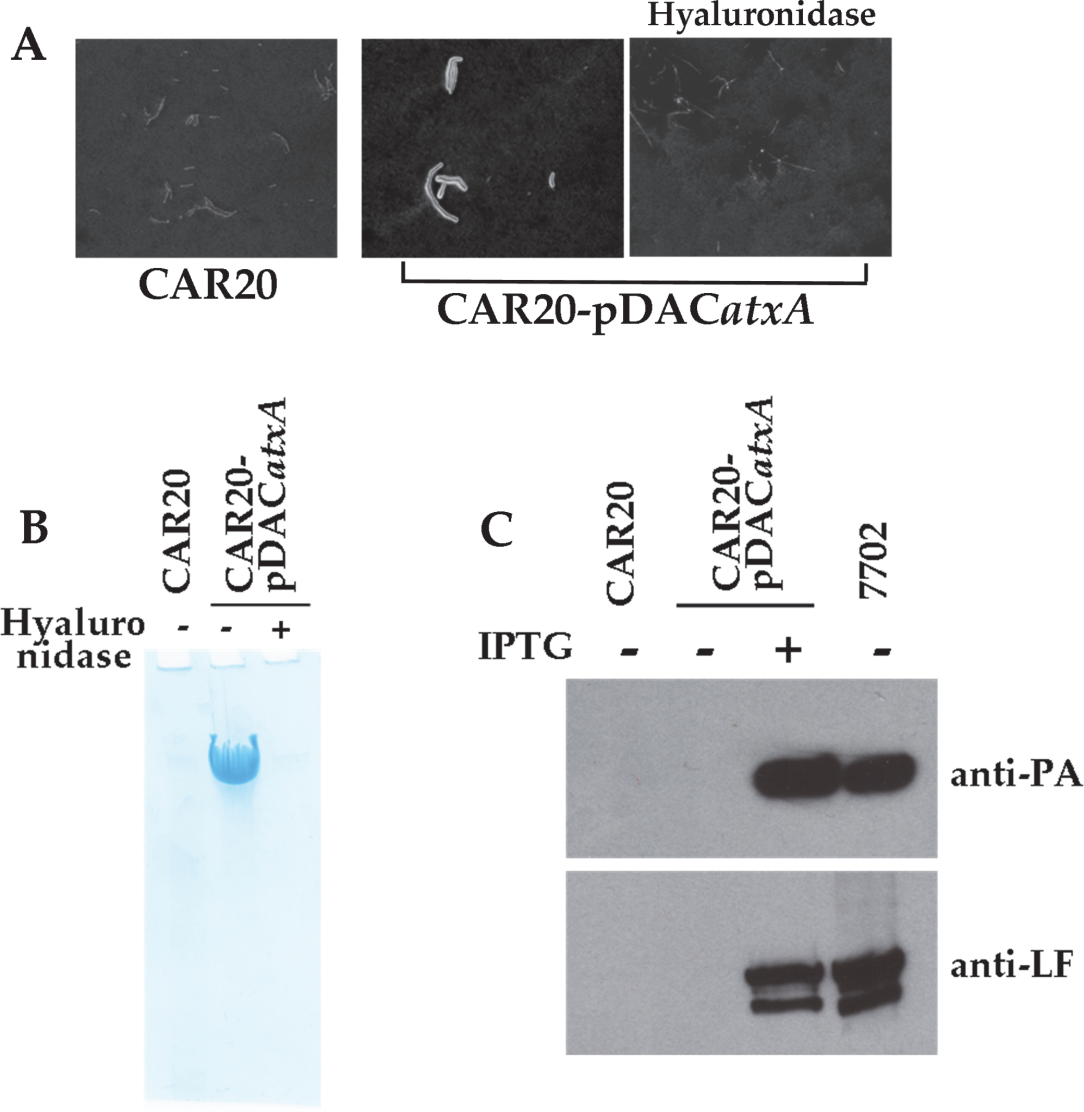
Expression of the HA capsule is regulated by AtxA. Complementation of the CAR20 strain with the pDAC*atxA* plasmid restores capsule and toxin expression after IPTG induction as observed by **(A)** India ink staining, **(B)** Alcian Blue staining of the bacterial culture supernatants, and **(C)** PA and LF toxin component production as described in Figs. 1 and 2 and in the Materials and Methods section. Hyaluronidase treatment confirms the HA nature of the capsule **(A,B)**.

We investigated further the mechanism of HA capsule regulation by examining the properties of a spontaneous rough mutant strain, CAR20, that was isolated in culture under 20% CO_2_. Multiplex PCR showed that this mutant strain still harbours pBCXO1. We examined the properties of this strain in inducing conditions: India ink staining revealed the absence of a capsule (Fig. 4A) and no band was detected through Alcian blue staining (Fig. 4B). DNA sequencing showed no mutation in the *hasACB* operon (S2A Fig.) that could account for this defect in HA capsule synthesis. However, analysis of toxin production showed that the non-encapsulated CAR20 strain was also unable to produce the toxin components PA and LF (Fig. 4C). These properties therefore strongly suggest that this strain carries a central defect that affects the regulation of PA, LF and the HA capsule. In *B*. *anthracis*, AtxA is the central regulator encoded by pXO1 that controls the expression of virulence factors under inducing environmental conditions. The *atxA* gene is also present on pBCXO1 ([20,24]); therefore, we hypothesized that AtxA is involved in *has* operon expression and may be defective in the CAR20 strain.

Comparison of *atxA* gene sequence of the CAR20 strain with that of the parental CAR strains showed three Single Nucleotide Polymorphisms at positions 153 (A to T), 157 (G to C) and 159 (C to A), and one base insertion at position 161 (T). These alterations result in amino acid changes at positions 51 (Leu to Phe) and 53 (Asp to Gln) and a shift of the reading frame after the 54th amino acid that introduces a stop codon (TGA) at position 77 (Table 5 and S3 Fig.). We confirmed the involvement of AtxA in HA-capsule production by complementation experiments. Introduction of a wild-type copy of the *B*. *anthracis atxA* gene into the CAR20 strain led to detection of *hasA* mRNA (S1B Fig.) and restored the production of the HA capsule (Fig. 4A & B) and of both PA and LF (Fig. 4C). Furthermore, the capsule in the complemented strain was completely digested by hyaluronidase, confirming that it was composed of HA (Fig. 4A & B). Our data thus show that HA-capsule expression in the *B*. *cereus* bv *anthracis* CA strain is regulated by AtxA.

**Table 5:** Characterisation of the spontaneous mutations in AtxA.

AtxA domains*	**WH**						**HTH**			**PRD1—PRD2**		
Codon position	6	51	53		54	74	104	141		286	322	332
**Mutation type** **	SNP	SNP	SNP	SNP	INS	SNP	INS	DEL	INS	INS	INS	VNTR
Base Change	T > C	A > T	G > C	C > A	T	C > A	A	A	A	A	A	ATTATA
**AA Change** ^§^	Ser > Pro	Leu > Phe	Asp > Gln		—	Pro > Thr	—	—	—	—	—	Tyr, Asn
Premature stop codon	—	+				—	+	+	+	+	+	—
Mutant	CAR2	CAR20				CAR4	CAR-P2	CAR3CAR-P5CAR-P6	CAR-P7CAR-P12	CAR-P3	CAR-P1	CAR1CAR5CAR-P8

*The AtxA domains were defined according to (Hammerstrom *et al*., 2011) WH, Winged Helix-turn-helix; HTH, Mga-like Helix-Turn-helix; PRD, PhosphoTransferase Regulation Domain.

**SNP, Single Nucleotide Polymorphism; INS, Insertion; DEL, deletion; VNTR, Variable Number Tandem Repeat

^§^AA, amino Acid

We consistently observed that rough mutants formed at the edges of smooth colonies after 48h of growth on capsule-inducing solid medium under 5% CO_2_. We screened fifteen spontaneous rough mutants of the CAR and CAR-P strains for LF production; none produced LF. Multiplex PCR analysis showed that two mutants had lost pBCXO1. We determined the sequence of the *atxA* gene in the remaining thirteen non-encapsulated mutants. All thirteen strains carried mutations in the *atxA* gene (Table 5 and S3 Fig.). Two mutants carried one base change leading to an amino acid substitution: either Ser to Pro (CAR2) or Pro to Thr (CAR4). These two mutations were located in the winged helix-turn-helix WH region in the N-terminal part of AtxA [49,50]. Three other mutations, located in the Phosphotransferase Regulation domain PRD2, resulted in either the addition of one (CAR-P8) or loss of one (CAR1, CAR5) of the two VNTRs (encoding the sequence Tyr-Asn) present in this region. The eight other *atxA* mutants had either a deletion or an insertion of a single base introducing a frameshift mutation. Six were located in the Mga-like helix-turn-helix HTH region; five of them affected codon 141, another insertion (CAR-P3) was located between the PRD1 and PRD2 domains, and another one (CAR-P1) was in PRD2 (Table 5).

### Respective roles of the HA capsule and toxins in virulence and dissemination

We found that despite the absence of pBCXO2—and thus of the PDGA capsule—in the CAR strain, this strain nonetheless exhibited high virulence both in mice and guinea pigs (Tables 3 and 4). Therefore, we investigated the specific contributions of the HA capsule and the toxins to the high virulence of the CAR strain. We constructed CAR-derived mutants lacking each of these virulence factors and examined their virulence when delivered by subcutaneous or intranasal routes in mice.

We inactivated the *pagA* gene in the CAR strain resulting in the non-toxinogenic CAR-P(Δ*pagA*) strain. This HA-encapsulated strain still retained virulence when delivered by the intranasal route in mice, and its virulence was only a 400-fold lower than that of the CAR strain (LD_50_: 10^7^ spores *vs* 2.5 x 10^4^, Table 3). In contrast, its virulence was highly attenuated in mice inoculated by the subcutaneous route, and was approximately 100,000-fold lower than that of the CAR strain (LD_50_: 6 x 10^7^ spores *vs* 550, Table 3).

Elimination of the HA capsule in the CAR strain (CAR-H(Δ*hasA*) strain, see above) led to a non-encapsulated strain (Figs. 1A & 2) that still produced PA and LF (Fig. 1B). This strain can thus be considered as equivalent to the *B*. *anthracis* Sterne strain. The CAR-H(Δ*hasA*) strain was avirulent when delivered by the intranasal route, as is the 7702 Sterne strain, (Table 3). However, this strain exhibited residual virulence when delivered by the subcutaneous route, with a virulence 5,500-fold lower than that of the CAR strain, but only 10-fold lower than that of the Sterne strain (Table 3).

Elimination of both virulence factors (toxins and HA capsule) either after loss of pBCXO1 (CAR-R strain) or through mutations inactivating the central regulator *atxA* (CAR20 strain), resulted in the absence of virulence in mice inoculated by the subcutaneous route (Table 3). This shows that no additional factor on the *B*. *cereus* chromosomal background significantly contributes to virulence of the CA strain in the absence of the toxins and the PDGA and HA capsules.

**Fig 5:**
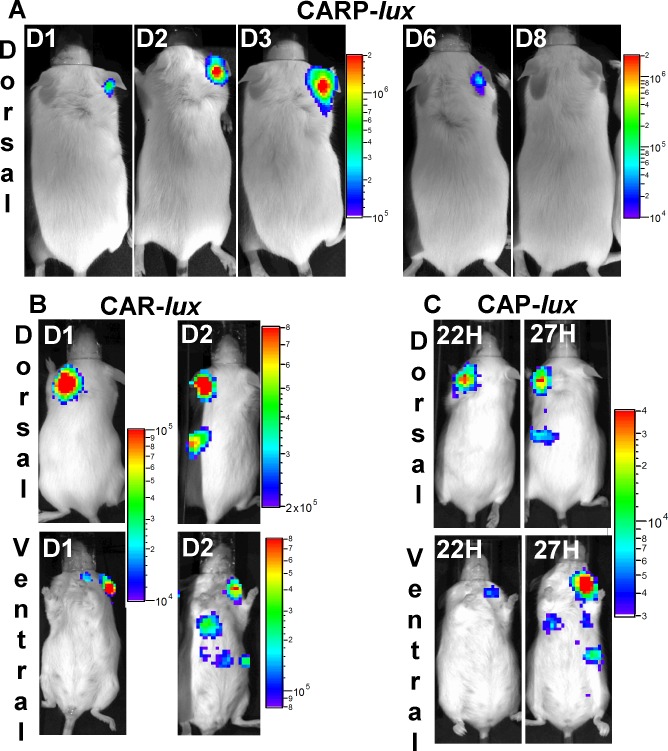
*In vivo* dissemination of the *B*. *cereus* bv *anthracis* CA strain during cutaneous infection in mice. Mice were inoculated into the ear pinna with spores of **(A)** CARP-*lux* (9 mice, inoculum 1 x 10^7^; all mice survived), **(B)** CAR-*lux* (8 mice, inoculum 1 x 10^5^; all mice died), or **(C)** CAP-*lux* (11 mice, inoculum 1 x 10^5^; all mice died) strains and bioluminescence was analysed at the indicated times after infection (D: days). These image series show a representative dorsal and ventral view of the same mouse for the various strains. Black and white photographs are overlaid with false-colour representation of luminescence intensity expressed in photons s -1 cm^2^ sr -1.

We investigated further the role of each virulence factor in bacterial colonisation and dissemination. Bioluminescent derivatives were constructed in strains expressing the HA-capsule only (CAR-P(Δ*pagA*)), the HA-capsule together with the toxins (CAR), and the HA capsule together with the PDGA-capsule(CAP(Δ*pagA*)). Mice were infected with these strains by cutaneous route in the ear pinna or by intranasal route. All mice that were cutaneously infected with a strain expressing only the polysaccharidic HA-capsule (CAR-P*lux* strain) exhibited a bioluminescent signal in the infected site (Fig. 5A) that was contained locally throughout the infection. The bioluminescent signal initially increased, but subsequently decreased and disappeared by day 8, after which no bioluminescence signal was detectable in the draining lymph node, spleen and lungs.

**Fig 6:**
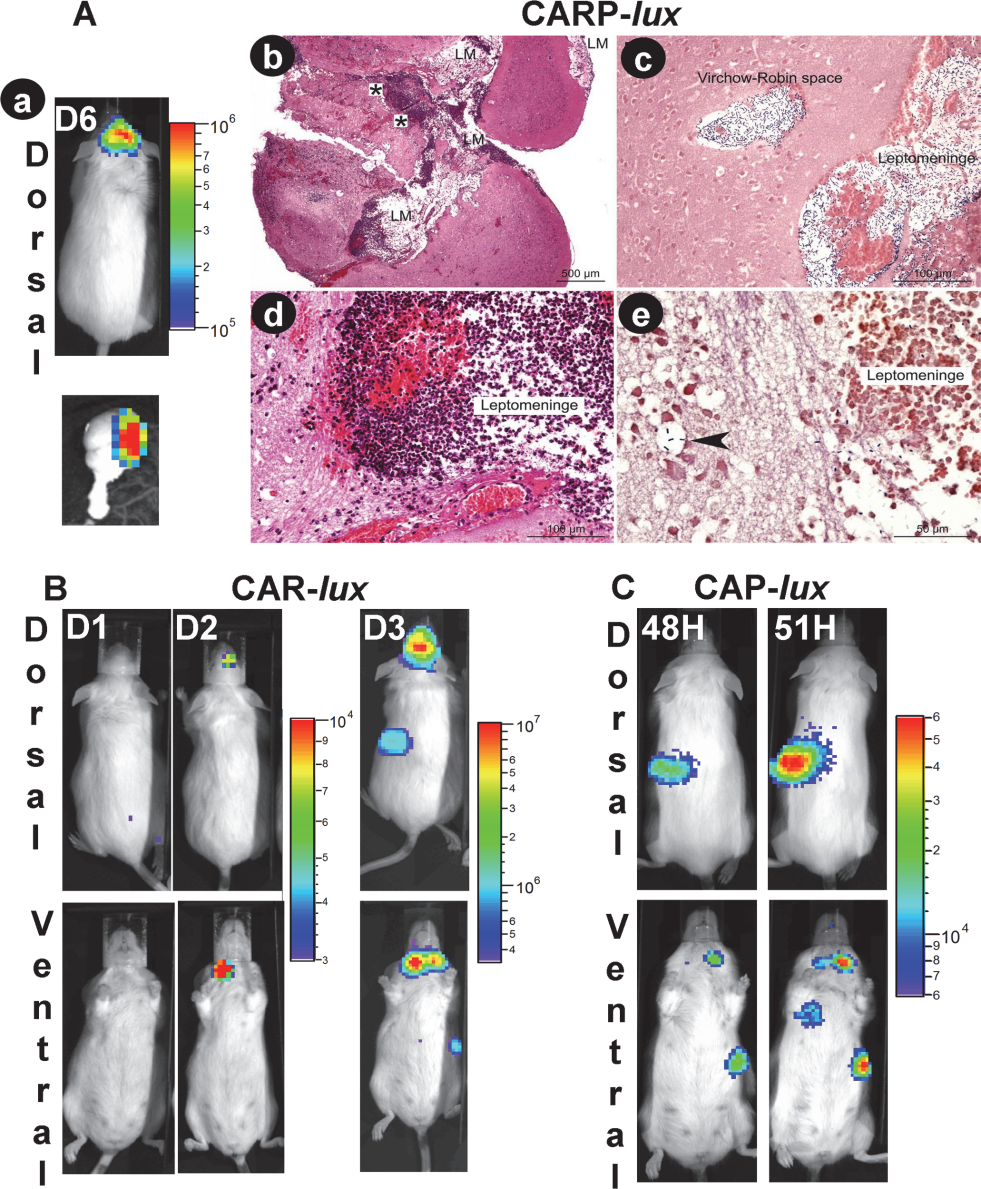
Local role of the HA capsule and *in vivo* dissemination of the *B*. *cereus* bv *anthracis* CA strain during intranasal infection in mice. Mice were inoculated intranasally with spores of **(A)** CARP-*lux* (13 mice, inoculum 1 x 10^8^), **(B)** CAR-*lux* (16 mice, inoculum 1 x 10^6^; all mice died), or **(C)** CAP-*lux* (14 mice, inoculum 1 x 10^6^; all mice died) strains and bioluminescence was analysed at the indicated times after infection as in Fig. 5 (D: days). (**Ab-e)** Histological characterisation of the infected brain tissue shown in **Aa**, bottom panel; **(Ab)** Diffuse inflammatory lesion centred on leptomeninges (LM), multifocally extending to the brain parenchyma (star); **(Ac)** high density of bacteria in the leptomeninges and Virchow-Robin spaces; **(Ad)** inflammatory infiltrates consisting of neutrophils, haemorrhages and oedema provoking a marked distension of leptomeninges and **(Ae),** at higher magnification, extending to the cerebral parenchyma with the presence of bacteria in the neuropil (arrowhead) highly suggestive of a rupture of the blood-brain barrier. **(Ab & d)**: HE staining; **(Ac & e)**: Gram staining.

Intranasal infection with 10^8^ spores of the CAR-P*lux* strain resulted in detection of a bioluminescent signal in five out of 13 animals (38%) in the dorsal region of the head. The bioluminescent bacteria remained restricted to this area throughout the infection (Fig. 6Aa) and no bioluminescence signal was detectable in the draining lymph node, spleen and lungs. All mice presenting a bioluminescence signal died in the 24h following the detection of the signal. Necropsy showed a bioluminescent focus of infection associated with the brain (Fig. 6Aa bottom) and the floor of the cranial chamber was bioluminescent. Histological analysis showed that the infection had disseminated to the central nervous system (Fig. 6Ab-e). A diffuse inflammatory lesion was centred on the leptomeninges and peripheral Virchow-Robin spaces (Fig. 6Ab). This lesion was characterised by haemorrhages, oedema, a marked infiltration of neutrophils (Fig. 6Ad), and a very high density of bacteria (Fig. 6Ac). The presence of multifocal extensions of inflammatory infiltrates in peripheral cerebral parenchyma, associated with a destruction of the neuropil and intra-parenchymal infiltration of bacteria (Fig. 6Ae) is highly suggestive of a multifocal rupture or permeability modification of the blood-brain barrier.

When the toxins were co-expressed with the HA-capsule (CAR*lux* strain), bioluminescent bacteria disseminated from the initial site of cutaneous infection, i.e. the ear pinna (Fig. 5B), to the draining lymph node, and then to the spleen and lungs just before death. Intranasal infection with the CAR*lux* strain resulted in the detection of a bioluminescence signal in the nasopharynx of 81% of the animals (9 out of 11); bioluminescent bacteria then spread to the draining cervical lymph node and the spleen (Fig. 6B). For two of the infected mice, no signal was detectable in the nasopharynx, although subsequent systemic dissemination was similar (S1C Fig.).

When both PDGA and HA capsules were co-expressed in the absence of toxins (CAP*lux* strain), bioluminescent bacteria initially multiplied at the site of cutaneous infection (the ear pinna), following which they spread to the draining lymph node, spleen and lungs (Fig. 5C). When bacteria were administered intranasally, no signal was detectable at the site of entry (nasopharynx), whereas local and systemic dissemination was essentially similar to what we observed during cutaneous infection, i.e. draining lymph node, spleen and lungs (Fig. 6C).

In conclusion, the bacterial pattern of colonisation and dissemination was profoundly modified when bacteria expressed either the toxins or the PDGA-capsule together with the HA-capsule.

### Efficiency of the FIS+PA vaccine in immunoprotection against infection with the *B*. *cereus* bv *anthracis* CA and CI strains

**Fig 7:**
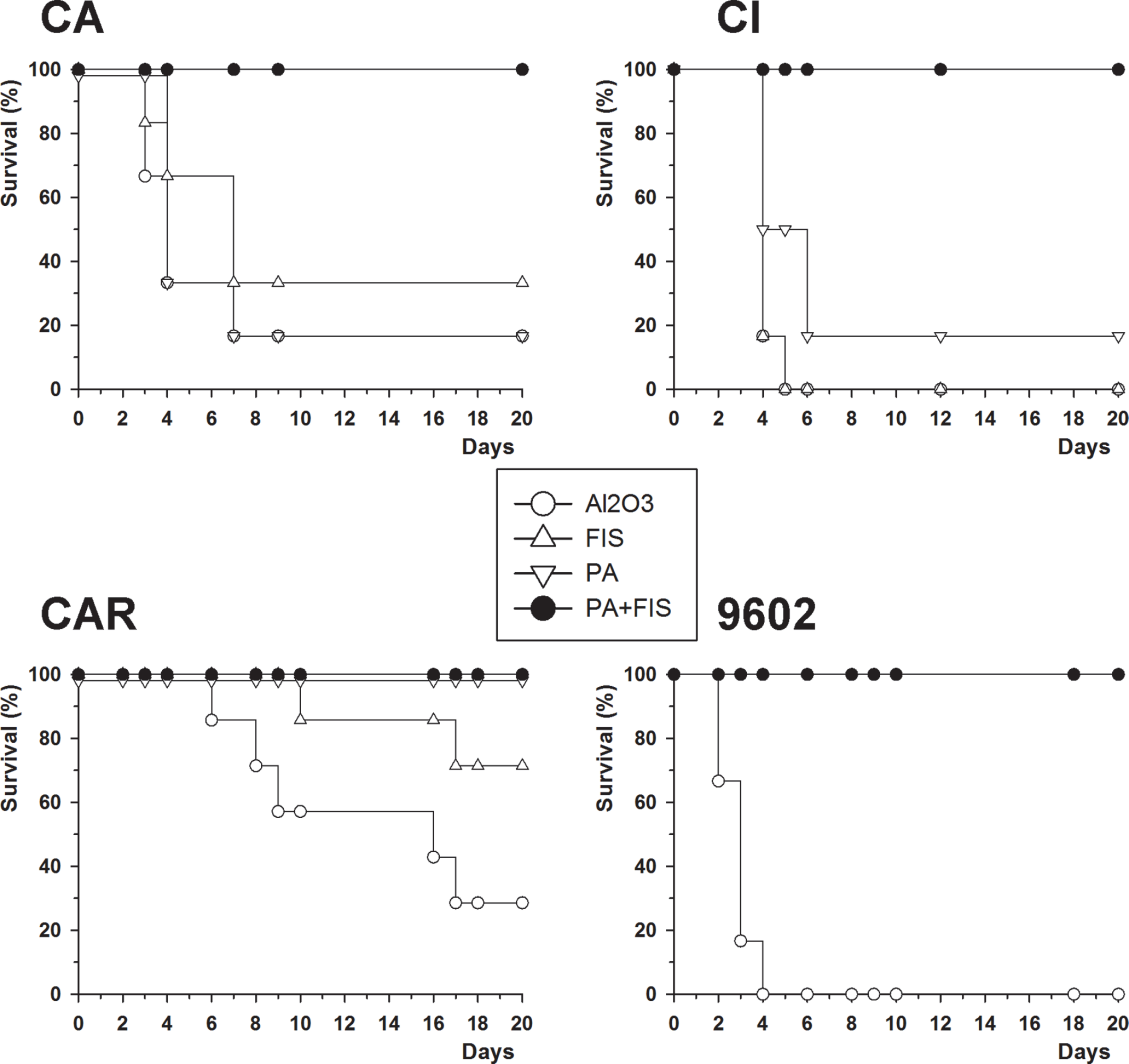
FIS+PA vaccine provides protection against subcutaneous challenge with the CA and CI strains in mice. Mice were immunised subcutaneously with rPA (10μg) and formaldehyde inactivated spores (FIS; 1 x 10^8^) at day 0 and 15, and were challenged with spores of the *B*. *cereus* bv *anthracis* CA (460 spores), CI (1150 spores), CAR (400 spores) or the *B*. *anthracis* 9602 (480 spores) strain as described in Materials and Methods. Survival was followed over a 20-day period. Results are representative of at least two independent experiments that gave similar results (six mice per group). Statistical significance was calculated by the log-rank test with GraphPad Prism software (*p* < 0.001; comparisons were made between the group immunised with PA+FIS *versus* that immunised with Al_2_O_3_ for each strain).

An important issue following the emergence of these strains is whether anthrax vaccines can protect against infection by these bacteria. We tested the efficacy of the formaldehyde inactivated spore (FIS) + PA (Protective antigen) vaccine ([42]) during sub-cutaneous infection of mice with spores of the *B*. *cereus* bv *anthracis* strains. We first tested immunoprotection against a challenge with spores of the fully virulent *B*. *cereus* bv *anthracis* CA and CI strains expressing both PDGA and HA capsules together with the toxins. Protection was afforded only when both FIS and PA were used in the immunisation regimen (Fig. 7), and no significant protection was obtained when FIS or PA was used alone. This finding is consistent with that reported for a fully virulent *B*. *anthracis* strain ([42], and Fig. 7). In contrast, when we challenged mice with the CAR strain that expressed only the HA-capsule and the toxins, immunisation with FIS alone led to partial protection and immunisation with PA alone led to total protection (Fig. 7).

## Discussion

Our study shows that the virulence of the pBCXO1- and pBCXO2-harbouring CA and CI strains is similar to that of wild-type *B*. *anthracis* in mice and guinea pigs inoculated either through cutaneous or inhalational routes. However, loss of pBCXO2—and thus of the PDGA capsule while retaining toxin production—did not affect the virulence by inhalational route and only moderately impaired it by cutaneous route (8- to 10-fold increase in LD_50_). This is in clear contrast with *B*. *anthracis*, where the loss of pXO2, as in the Sterne strain, leads to significant attenuation. Indeed, intranasal delivery of the Sterne strain in immunocompetent mice is associated with an absence of virulence, while cutaneous infection results in significant attenuation (>1,000-fold) in mice and guinea pigs [7,46,47].

Here we show that the CA and CI strains also possess a hyaluronic acid (HA) capsule that plays a significant role as an additional virulence factor. It is encoded by the *has* operon, located on pBCXO1, that we show to be functional both in the CA and CI strains, in contrast to *B*. *anthracis* that contains a non-functional *hasA* gene [8,19]. Our analysis of the available genome data for the pathogenic encapsulated *B*. *cereus* 03BB102 strain also revealed a wild type *hasA* gene, as reported for the *B*. *cereus* G9241 strain [18].

The *B*. *cereus* bv *anthracis* CA and CI strains are the first examples in which the PDGA capsule coexists simultaneously with another type of capsular structure. We also demonstrate that CO_2_/bicarbonate and the central regulator AtxA are necessary for synthesis of the HA capsule, similar to their requirement for the expression of the PDGA capsule in *B*. *anthracis*. Expression of the *has* operon is strongly upregulated in *B*. *cereus* G9241 under CO_2_/bicarbonate conditions [51]. We implicate AtxA in this process by isolating spontaneous mutants in *atxA* in the *B*. *cereus* bv *anthracis* CAR/CAR-P strains; these mutants were avirulent, devoid of capsule and did not produce toxins. Complementation with a wild-type *atxA* gene led to restoration of capsule and toxin production. Thus, the *has* operon, located on pBCXO1 outside the pathogenicity island [8] may be assigned to the AtxA regulon in *B*. *cereus* bv *anthracis*. The *atxA* mutations were mainly located in the domains that are reported to be potentially involved in DNA binding and phosphotransferase regulation. These domains have also been described in MgA, which is a global regulator of virulence in group A *Streptococcus* [49,50]. *AtxA* gene expression is high in CO_2_/bicarbonate culture conditions [51]. Dimerisation of AtxA in these conditions leads to high AtxA activity and upregulation of the AtxA regulon [49]. *AtxA* mutations occurred at a high frequency in CAR/CAR-P strains grown under these conditions. Noteworthy, occurence of such *atxA* mutations was not observed in *B*. *anthracis*, using bioluminescence as a marker of AtxA-dependent gene expression with the P*pagA-lux* 9602R-derived bioluminescent strain [52].

Under CO_2_/bicarbonate conditions, the expression of numerous genes on the chromosome and pXO1 is upregulated to a higher extent in the pathogenic *B*. *cereus* G9241 strain than in the *B*. *anthracis* Sterne strain [51]. Therefore, it could be proposed that high expression of AtxA-controlled genes under CO_2_/bicarbonate conditions in the *B*. *cereus* CA strain has a deleterious effect on bacterial growth that may lead to the selection of *atxA*-inactive mutants. Genes belonging to the Atxa regulon, and more generally CO_2_/ bicarbonate regulated genes, [51,53,54] clearly need to be further characterised in *B*. *anthracis* and the emerging pathogenic *B*. *cereus* CA and CI strains, especially as these *B*. *cereus* strains harbour the pBCXO1 and pBCXO2 plasmids in a different chromosomal background.

The HA capsule strongly contributed to virulence in the absence of the PDGA capsule. Bacteria of the toxinogenic pBCXO1^+^ CAR(Δ*hasA*) strain in which the *has*A gene was inactive were avirulent in animals inoculated by inhalational route and their virulence was also strongly attenuated in animals inoculated cutaneously. The virulence of this CAR-H(Δ*hasA*) strain was not however, equivalent to that of the *B*. *anthracis* pXO1^+^ Sterne strain, although both strains express the toxins without any capsule. Virulence was 7.5-fold more attenuated for the CAR-H strain than for the Sterne strain, which may be related to differences in genetic background, regulatory networks or phenotypic properties that need to be addressed. Our study, and the clinical context in which the pathogenic G9241, 03BB102, 03BB87 and Elc2 *B*. *cereus* strains were isolated, therefore highlight the potential role of a polysaccharide capsule as a virulence factor. This factor may provoke fatal anthrax-like pulmonary infections in humans, as reported in US metal workers [13,14].

The CARP strain (HA+Tox-) does not produce toxins but retains HA-capsule expression. The virulence of these bacteria was highly attenuated in mice inoculated either cutaneously or intranasally. However, attenuation was less pronounced when bacteria were delivered by inhalational (500-fold) than by the cutaneous route (100,000-fold). Interestingly, the residual virulence of the HA+Tox- strain delivered by inhalational route is associated with a different mechanism of dissemination than that reported for a PDGA-encapsulated non-toxinogenic *B*. *anthracis* strain [30]. We used a HA+Tox- bioluminescent derivative to examine bacterial dissemination *in vivo*. We show that bacterial multiplication remains local and that dissemination occurs from the nasopharynx to the adjacent brain, leading to lepto-meningo-encephalitis and death. The cribriform plate of the ethmoid bone has been suggested to be involved in the dissemination of pathogens present in the nasal cavities such as bacteria, virus and parasites along the olfactory nerves to the olfactory bulb and the meninges and subarachnoid space [55,56,57,58]. One may speculate whether this could be the case during infection with the HA+Tox- CARP mutant bacteria.

These observations show that the HA capsule may lead to a more successful infection and may provide a survival advantage for the bacterium when it enters the infected host by the respiratory route. Group A *Streptococcus*, a major human pathogen, also produces a HA capsule that is involved in the colonisation of the upper airways [59]; this capsule is a virulence factor encoded by a similar *has* operon in response to host environmental signals [60].

Toxin expression was essential for enhanced virulence in the presence of the HA capsule (CAR strain). Indeed, bacteria expressing both the HA-capsule and toxins showed a profoundly different pattern of infection to that of the HA+Tox- strain. Intranasal delivery of the CAR strain was associated with local bacterial multiplication in the nasopharynx, followed by local and systemic dissemination, and death. Similarly, during cutaneous infection, the bioluminescent HA+Tox- strain remained localised to the initial infected site, whereas co-expression of the toxins with the HA capsule enabled efficient local and systemic bacterial dissemination leading to death. Similarly, toxin expression in the presence of the PDGA capsule (and in absence of the HA capsule, CA-HΔ*hasA* strain) led to systemic dissemination; no signs of neuropathological involvement were observed during infection with this strain. To note, this strain could be considered as an equivalent of wild-type *B*. *anthracis*, expressing toxins and the PDGA capsule: dissemination to the brain was never observed in the mouse model of infection [30,45]. This effect of the toxins in promoting systemic dissemination is similar to what has been reported for *B*. *anthracis* [45,61].

We confirmed the synergistic action of the toxins and the HA-capsule in virulence through immunisation/vaccination experiments. Immunisation with PA alone was sufficient for the host immune defences to control the infection by the HA+Tox+ CAR strain. Once the toxins are neutralised, the HA capsule does not by itself lead to the successful spread of infection. Thus, a polysaccharide capsule does not impair the protection afforded by a PA-based vaccine, as also shown for the *B*. *cereus* G9241 strain that produces two types of polysaccharide capsules [62].

Our study also shows that co-expression of the PDGA capsule with the HA capsule in the CAP strain leads to higher virulence than that of a HA-only encapsulated CARP strain. The LD_50_ of this strain in mice was close to that of the parental CA and CI strains and dissemination was similar to that of *B*. *anthracis* [30,52,63]. These data confirm the role of the PDGA capsule in the extreme susceptibility of mice to *B*. *anthracis* infection [7,42,64]. Furthermore, as expected [42], immunisation with PA alone did not result in immunoprotection in the mouse model of infection with this PDGA-expressing HA-encapsulated *B*. *cereus* strain. Addition of inactivated spores (FIS) to a PA-based vaccine was necessary to achieve total protection, similar to what is required to protect against *B*. *anthracis* infection in mice [42]. Presence of a HA-capsule thus does not alter the efficacy of the PA+FIS vaccine against infection with the *B*. *cereus* bv *anthracis* strains. Therefore, the PA+FIS vaccine should provide a similar degree of protection against both *B*. *cereus* bv *anthracis* infection and *B*. *anthracis* infection, in case this emerging pathogen should disseminate in either animals or humans.

The *B*. *cereus* bv *anthracis* CA and CI strains represent a unique example of strains that may be considered as at the borderline between *B*. *cereus* and the monomorphic *B*. *anthracis*. Indeed, these strains display some characteristics thus far found only in *B*. *anthracis* and others that are restricted to *B*. *cereus* strains. Similar to *B*. *anthracis*, the CA and CI strains (i) express toxins and a PDGA capsule both regulated by AtxA; (ii) harbour pBCXO1 and pBCXO2 plasmids that are very close to the *B*. *anthracis* pXO1 and pXO2 (iii) show a similar virulence to that of *B*. *anthracis* both in mouse and guinea pig models of infection; and (iv) have a pattern of dissemination that resembles that of *B*. *anthracis* [30]. Moreover, they also share a "PlcR-null" phenotype with *B*. *anthracis*, as PlcR-controlled enzymatic activities are not detected in the CA and CI strains [20]. This contrasts with the great majority of *B*. *cereus* strains which possess a functional PlcR [2], and also with the pathogenic pBCXO1-harbouring strains [13,14,16]. However, the *plcR* mutation in the CI and CA strains ([24] and S2B Fig. respectively) is different from that considered as characteristic of the *B*. *anthracis* lineage. This mutation is present in all strains of *B*. *anthracis* thus far analysed, and is currently considered as a hallmark of the *B*. *anthracis* lineage [6,65]. The *B*. *cereus* bv *anthracis* strains CA and CI also present some properties that associate them with pathogenic *B*. *cereus* strains: they belong to the same phylogenetic group as *B*. *cereus* [2,24] and both *B*. *cereus* bv *anthracis* and pathogenic pBCXO1-harbouring *B*. *cereus* strains express a HA capsule, due to a functional *hasACB* operon. Interestingly, *in silico* analysis on 163 sequenced *B*. *anthracis* genomes (Sylviane Derzelle, Guillaume Girault; personal communication) shows that the *hasA* mutation is present in *B*. *anthracis* strains of both the A and B main branches. Therefore, the *hasA* frameshift mutation may represent another characteristic of the *B*. *anthracis* lineage, and thus may be the first to be located on the plasmid pXO1.

If we consider the origin of the pBCXO1/pXO1-like plasmids, it is noteworthy that the pathogenic G9241, 03BB102, 03BB87 and Elc2 *B*. *cereus* strains were isolated from patients living in rural zones of the same geographical area (Texas and Louisiana) where anthrax is endemic [13,14,16]. However, the absence of the *hasA* mutation characteristic of *B*. *anthracis* makes it unlikely that they acquired the plasmid from *B*. *anthracis*. Some environmental *B*. *cereus* strains isolated in the same area are very similar to 03BB102, but are devoid of pBCXO1 [2,13] These strains may play the role of recipient host during horizontal transfer with a still unknown bacteria harbouring a pBCXO1 ancestor. An equivalent of the pBCXO1 must exist in Africa, because it is present in the *B*. *cereus* bv *anthracis* CA and CI strains.

Our data show that acquisition of a pXO1-like plasmid carrying a functional *has* operon together with the toxin genes and the AtxA regulator clearly gives a selective advantage to the recipient *B*. *cereus*. This event enables successful infection of the mammalian hosts, which would lead to an increase in the bacterial biomass and to subsequent environmental contamination. Further amplification cycles would then favour pathogen emergence and spread.

*B*. *anthracis* is thought to have evolved as a monomorphic lineage within the *B*. *cereus* group, with a strict co-evolution between the chromosome and the two plasmids pXO1 and pXO2. The *B*. *cereus* bv *anthracis* CA and CI strains may be considered as a new lineage among the *B*. *cereus* group, that appeared through an ongoing co-evolution process similar to the emergence of *B*. *anthracis* during the holocene [2,65]. It is tempting to speculate that pBCXO1 is an ancestor of the *B*. *anthracis* pXO1, which further evolved with pXO2 and the chromosomal genetic background.

## Supporting Information

S1 Table Oligonucleotides used for sequencing. (DOC)

S2 Table Accession numbers for genes used in this work. (DOC)

S1 Fig A. Multiplex analysis of the pBCXO2-cured CAR and CAR-R strains: absence of pBCXO1 in the CAR-R strain; *pagA*, *lef*, *cya* genes are on pBCXO1, *capB* is presnet on pBCXO2. **B.** Complementation of the CAR20 strain with the pDAC*atxA* plasmid restores *hasA* mRNA expression with *gyrB* as positive control. **C.** A mice representative of the two mice infected intranasally with the CAR strain (same conditions as in Fig. 6B) displaying no bioluminescent signal in the nasopharynx, but similar systemic dissemination in spleen and lungs at 96h.
(TIF)

S2 Fig A. Sequence of the *hasACB* operon in the CAR and CAR20 strains; both sequences are identical to the one published for the CI strain [24]. B. Sequence of the *plcR* gene in the CA strain; it is identical to the one published for the CI strain [24].
(DOC)

S3 Fig Sequences of the *atxA* gene in the CAR20, CAR- and CARP-derived spontaneous rough mutants. Mutations are indicated and the corresponding premature stop codon is identicated as underlined when occurring. The sequences are compared to that of the *B*. *anthracis* Ames strain.
(DOC)
