# Correction: Capsules, Toxins and AtxA as Virulence Factors of Emerging *Bacillus cereus* Biovar *anthracis*

**DOI:** 10.1371/journal.pntd.0003746

**Published:** 2015-04-22

**Authors:** The PLOS Neglected Tropical Diseases Staff

The first three authors, Christophe Brézillon, Michel Haustant, and Susann Dupke, should be noted as contributing equally to this work. The last two authors, Silke R. Klee and Pierre L. Goossens, should be noted as joint senior authors of this work.
