# Supplementary material for: Capsules, Toxins and AtxA as Virulence Factors of Emerging Bacillus cereus Biovar anthracis
Source: PLoS Negl Trop Dis. 2015 Apr 1;9(4):e0003455. doi: 10.1371/journal.pntd.0003455 (PMC4382292; doi:10.1371/journal.pntd.0003455)
Supplement: S1 Table — (DOC) [file pntd.0003455.s001.doc]

**Table S1:** Oligonucleotides used for sequencing

| hasACB operon | | Sequence |
| --- | --- | --- |
| hasAFa | CCGCAGTATGACAGTGTAGGATTATATTTG | |
| hasARa_498 | TGTAGCAGCAATTTCTCGTTGAGTTCTAAG | |
| hasAF1_205 | GGGATATATGGAACGCTGATGGTGATTTAC | |
| hasAR1_627 | GGTAACAATAGCATCTGCTGTTGTCCG | |
| hasAFb_625 | ACCATTGATTCAGATGGTGATTTGTTCCCA | |
| hasARb_1099 | ACCAATGCCAAGTGAAATTAAACTTTCTCT | |
| hasAFc_892 | GGAAGTCAGATGTTCCTTGGTGAGGAGGTG | |
| hasAR2_1395 | ACGTGTTCCCCAACCATTAGATTTAATAGT | |
| hasCF2_31 | GCGGGATTAGGCACAAGATTTTTACCC | |
| hasCR1_333 | ATGTCCTAATCCTTTAGGTTCTTTTTGCCG | |
| hasCFA_304 | CGGCAAAAAGAACCTAAAGGATTAGGACAT | |
| hasCR2_879 | GATAAGTTTATCTCGAGTGATATTTCTCAA | |
| hasCF_766 | GTAGGGGATAAATTTGGCTTTATTAAAGCG | |
| hasBF1_19 | GGAACAGGCTATGTTGGCTTAGTAACAGGT | |
| hasBR1_573 | TGGTGTACCAAAGGGTTTATATACTTCTTC | |
| hasBFb_511 | GGATCGGAAAATAAAACATCGGCAAATGTT | |
| hasBRb_1050 | TGGGTCATATGCAATCACCTGTGCACCTTC | |
| hasBFc_798 | GCGTTGGTTAAAGTTTCTGAAAGTTTACAA | |
| hasBR2_1329 | AGCTGTTATCTCTCCTTTTACTTCTCT | |
| atxA sequencing | Sequence | |
| atxA4F | GGATTCCTAACACCGATATCCATCGAAAAGGAACATATAAG | |
| atxAGSP2R | CGCCGAAAGAGTTTTGTAGCTC | |
| atxA579F | GTACACCTATTCAAAACACAAATTG | |
| atxA1440R | AAGCTTGGGCATTTATATTATCTTTTTGATTTCATGAAAATCTCTTTCTGTAGG | |
| axP2LRT | GCATTCTTGACCAGTTGGATT | |
| axP1RRT | TTTTCGATGGATATCGGTGTT | |
| atxAMidF | CGGAATTTCCTACAGAAAGAGATTTTCATG | |
| atxA3R | GTACCAGGCTTTCCGCTTCCTTCAGCCC | |
