# Supplementary material for: Capsules, Toxins and AtxA as Virulence Factors of Emerging Bacillus cereus Biovar anthracis
Source: PLoS Negl Trop Dis. 2015 Apr 1;9(4):e0003455. doi: 10.1371/journal.pntd.0003455 (PMC4382292; doi:10.1371/journal.pntd.0003455)
Supplement: S2 Table — (DOC) [file pntd.0003455.s002.doc]

**Supplementary Table 2 :** Accession numbers for genes used in this work

*B. cereus* bv *anthracis* CI plasmid pCI-XO1: NC_014331.1

*B. cereus* bv *anthracis* CI *atxA* (BACI_pCIXO101370)

*B. cereus* bv *anthracis* CI *hasACB* (BACI_pCIXO101130, BACI_pCIXO101140, BACI_pCIXO101150)

*B. cereus* bv *anthracis* CI *capB* (BACI_pCIXO200640)

*B. cereus* bv *anthracis* CI *gyrB* (BACI_c00050)

*B. cereus* 9241 *hasACB* (BCE_G9241_pBCXO1_0108 to 0110)

*B. anthracis* Ames Ancestor *hasACB* (GBAA_pXO1_0128 to 0130)

Vollum *hasACB* (BantV_010100028932; BantV_010100028937; BantV_010100028942)

Vollum *capB* (BantV_010100029487)

Vollum *gyrB* (BantV_010100026139)
