# Supplementary figures and images for: Capsules, Toxins and AtxA as Virulence Factors of Emerging Bacillus cereus Biovar anthracis
Source: PLoS Negl Trop Dis. 2015 Apr 1;9(4):e0003455. doi: 10.1371/journal.pntd.0003455 (PMC4382292; doi:10.1371/journal.pntd.0003455)

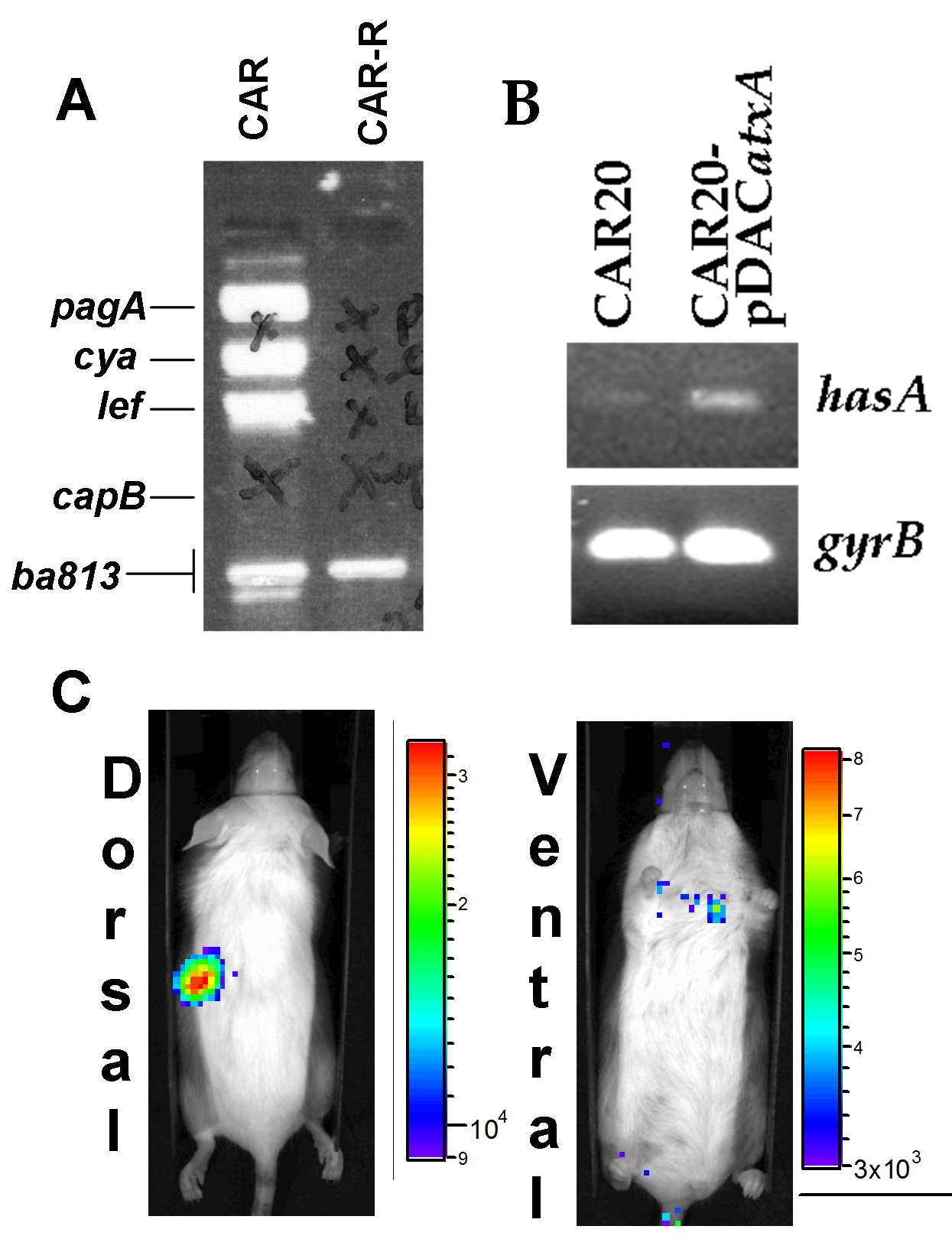

Supplement: S1 Fig — B. Complementation of the CAR20 strain with the pDACatxA plasmid restores hasA mRNA expression with gyrB as positive control. C. A mice representative of the two mice infected intranasally with the CAR strain (same conditions as in Fig. 6B) displaying no bioluminescent signal in the nasopharynx, but similar systemic dissemination in spleen and lungs at 96h. (TIF) [file pntd.0003455.s003.tif]
