# Supplementary material for: Capsules, Toxins and AtxA as Virulence Factors of Emerging Bacillus cereus Biovar anthracis
Source: PLoS Negl Trop Dis. 2015 Apr 1;9(4):e0003455. doi: 10.1371/journal.pntd.0003455 (PMC4382292; doi:10.1371/journal.pntd.0003455)
Supplement: S2 Fig — B. Sequence of the plcR gene in the CA strain; it is identical to the one published for the CI strain [24]. (DOC) [file pntd.0003455.s004.doc]

**Figure S2 :**

**A.** *B. cereus* CAR / CAR20 : *hasACB* operon sequence

ATGAAAAATTTTTTTATAAATATTAAACCTTATACGTCCTATGAGAAATTTAGATCGTATTTACCCAAAGGAGAAAATAGTTTAAAGCAAAATAAAATAAAATCTTCTTTAAAAATGCCCTTCATATTACTTGTTTTTTCTTTATTGATTGTAGCAATGTACTATATTAACTTTACCATTCGTGGAAGTTTTAGTTTGTTTTTAGGGATATATGGAACGCTGATGGTGATTTACTTACTTGGAAAACAATCATTATCATTTTTTTATCGACCAATTACAGGTGATAAGGTTCCAAATATGAAAGTGGCAGTAGTGGTCCCCTCATATAATGAAAGTGCAAGTGCTATTGTTAATACAATTAATAGCGTTTTAGCTCAAGATTATCCAATTCATGAAATTTTCTTTGTTGATGATGGTAGTAAGGATAAATCGGCTTATGAAGTAGCACTTAAAATGAGGGAGGAACTTCTTAGAACTCAACGAGAAATTGCTGCTACAACTAAGAATAATTGTTCTGAAATATTAGGTATTCCTGACTTAATCGTACATCGTTTACCTAAGAATTGCGGGAAAAGACATGCTCAATTATGGGCTTTTAAACGGACAACAGCAGATGCTATTGTTACCATTGATTCAGATGGTGATTTGTTCCCAAATGCTGTTAGAGAGTTATTGAAACCCTTTAATGATGAAAAAGTAATGGCCACAACTGGTCACGTGAACATTCGTAATAGAAATGATAATTTATTAACAAAACTAATTGATATGCGTTATGACAATGCGTTCCGTGTGGAGCGTGCAGCACAGTCCGTAACAGGAAATGTTCTTGTTTGTAGTGGGCCGTTAAGTTGTTATCGTAGAGAAGTAATAACTGAAAATTTAGAACATTATGGAAGTCAGATGTTCCTTGGTGAGGAGGTGCAGTTTGGAGATGATAGATGTCTAACTAATTATGCTATTTTGAAAGGGAAAACAGTTTATCAATCCACTGCTCGATGTATTACTGATGCTCCAACTACATTAAAACAATTTCTTAAACAGCAACTACGTTGGAACAAGTCATTTTTTAGAGAAAGTTTAATTTCACTTGGCATTGGTATGAAAAAACCAAATGTTCTTGTTTGGACAATTTTCGAAATATCGTTATGGATTTTATTTGGGCTTTCCCTACTTCTAAGTATTATTCTCAAGGCAAGTCATGTAGGGTTAATTTTGGCTGTTTATTATTTGGGTTATATTTCGTTAGCTGCATATGCTAGAAATGTATTTTATCTATTAAAACATCCCCTTACTTTCTTACTGGCGCCATTATATGGAATTCTCCATGTATTAGCACTATTACCTATACGCTTTTATGCTTTACTAACTATTAAATCTAATGGTTGGGGAACACGTTAATTACAGTAATTTTATGTATTTTTTTTAGGAGGATATTATTAAGTGAAGATTAGAAAAGCGATTATCCCAGCAGCGGGATTAGGCACAAGATTTTTACCCGCAACAAAAGCACAACCTAAAGAAATGTTGCCAATTGTAGATAAGCCAACTATTCAATATATTGTTGAAGAAGCTGTTAGCTCAGGAATAGAGGATATTATTATTGTAAGTGGGAGAGGGAAGCATGTTATAGAAGATCATTTTGATAAATCTTATGAGCTAGAACAAACTTTATTTAAGAAAAACAAAATAAAGACTCTTGAAGACATTGAATGCATTTCTAATTTAGCGAATATCCATTATATTCGGCAAAAAGAACCTAAAGGATTAGGACATGCTATATATTGTGCTAGACGTTTTATAGGTGAAGAACCTTTTGCAGTTTTACTTGGTGATGATATTGTTAGCTCTACGTATCCATGTCTCAAGCAATTGATAGATGTTTATGAGGAACATCACTGTTCAGTGGTAGGTGTTCAAAGGGTATTAGAAACAGAAGTGTCTAAATATGGAATAGTTAAATCGGCAAATCAAAATGTTAATCAATCTATTATTCCTATTTCTATGCTAGTTGAAAAACCACCTCTGGAAACTGCACCTTCAAATTTAGCTATAATGGGCAGATATATACTAAAACCAGATATTTTTGAGGTGTTAAAAAATTTACCTGTCGGATCAGGTGGAGAAATTCAGTTAACCGATGCAATCAATGTTTTAAATAAACAACAAAAAGTACTCGCCTTTGAATTTGATGGGAAAAGATATGATGTAGGGGATAAATTTGGCTTTATTAAAGCGACTATAGATTTTGCACTTCAGAGAGAAAGTCTAAAAGAAGATGTTTTGAGTTATTTGAGAAATATCACTCGAGATAAACTTATCAATAAATAAAATATGTGAAAAAGTGAGGAAGTCTTAATGAATATATCTATAGTAGGAACAGGCTATGTTGGCTTAGTAACAGGTGTGTGTCTATCAGAAGTAGGTCATAACGTCACTTGTATTGATATAGACGAAGAAAAAGTAAAAAAAATGAAATTGGGTTATTCACCAATATTTGAGCCATGTTTAGAGGAATTAATGAAGAATAATATTATAAAGGGAAGATTACATTTTACAACTAACTATGTTGATGGAGCTGATGGGGCAGAAATTTTTTATATTGCAGTTGGTACACCACAAAAAGAGGACGGTTCAGCCGATTTAAGCTTTATTAAGCAAGCAGCTATTAATATTGCTCGTACAATTAAGAATGATGTTATTATTGTGGTAAAAAGTACCGTGCCTGTAGGCACCAATATATATATTAAGAATCTTATTTTGAAAAATTTAAATTATGATGTAAAAGTAGATATTATTTCAAATCCAGAATTTTTACGTGAAGGTTCAGCTGTAAATGATACTTTTTATGGGGATCGTATTGTAATTGGATCGGAAAATAAAACATCGGCAAATGTTATGGAAGAAGTATATAAACCCTTTGGTACACCAATATTCAAAACAGATATCCAAAGTGCCGAAATGATAAAGTATGCGTCTAATGCATTCTTAGCCACAAAAATTAGTTTTATTAATGGGATAGCGAATTTATGTGAAATGGTTGGAGCAGATGTGGAAAAGGTAGCACAAGGGATGGGACAAGATAAAAGAATTGGTTCTCAATTTTTAAATGCAGGAATAGGATACGGTGGATCATGTTTTCCTAAAGATACACATGCGTTGGTTAAAGTTTCTGAAAGTTTACAACATAAATTTCATCTTTTAGAATCTGTTATTAAGCTGAATAAAAATCAACAAACAGTTTTGATAGAAAAAATAAAAAAACGTTTTGGAAGTATTGTTGGCAAAAAAATTGCTTTATTAGGACTTTCTTTTAAACCGAATACGGATGATTTAAGAGAAGCTCCCTCTATTCCCATAGCAAGAAAATTAGTAGAAGAAGGTGCACAGGTGATTGCATATGACCCAGTTGCAATTAAGAACGCGCGAGAAGTGTTGCCTAAAGAGGTACATTATGTATATTCTACTATGGAGGCTCTTACTGAAGCAGACATTGCCTTAGTTCTAACAGAGTGGGATGAAGTGGTCGATTCTTTACTATTAAAAGCAAGTCAATTAATGAAAGAACCCGTAATTTTTGATGGTCGAAATTGTTTCGAACTTAATGAGGCTAAAAATTACGATGTTGAATACCACTCGATTGGAAGACCCTCAGTTTTAAGAGAAGTAAAAGGAGAGATAACAGCT

**B.** *B. cereus bv anthracis* CA : *plcR* sequence

ATGCACGCAGAAAAATTAGGAAATGAAATTAAGAAAATTAGGACGATGAGAGGATTAACACAAAAACAGTTATCCGAGAA

CATATGTCATCAATCGGAAGTGAGTAGAATTGAATCGGGCGCGGTATACCCAAGTATGGATATATTGCAAGGTATCGCAG

CAAAATTACAAGTTCCCATTATTCATTTTTATGAGGTACTCATTTATTCTGATATTGAGAGGAATAAGCAGTTAAAAGAT

CAAATTATTATGCTTTGTAAGCAAAAGAAATATAAAGAAATTTATAATAGAGTATGGAATGAGTTGAAAAAGGAAGAATA

TCACCCCGAGCTTCAGCAATTTCTTCAATGGCAATATTATGTAGCTGCTTACATATTGAAAAAAATCGATTACGAATATT

GTATTTTAGAATTAAAGAAATTGCTCAATCAACAATTGGCAGGAATAGATGTATATAAGAGTCTTTATATTGAAAACGCA

ATTGCAAACATTTATGCTGAAAATGGCTATTTGAAGAAGGCTATTGATTTATTTGAAAATATATTAAAACAATTAGAGGC

ATTGCATGATAATAAAGAATTTGATGTGAAGGTGAGGCATAATCATGCAAAAGCATTATTCTCAGATAATCAATACGAAG

AAGCGCTTTGTCACGCAAATAGAGCTATTGAACTATCGTGTCAAATTAATAGTATGACATTAATTGGACAGTTATACTTT

CGAAAAGGTCAATGCCTAGCAAAGCTAGGGTGTGATAGAGCGGAAATTGAAGATGCTTACGAAAAAGCGTGCTTCTTTTT

TGATATATTAGGAAACCATACGTTAAAAGAATCAATTATAAAAAAAAATGAAGAAATGAAAAGGACCTAA
