# Supplementary material for: Capsules, Toxins and AtxA as Virulence Factors of Emerging Bacillus cereus Biovar anthracis
Source: PLoS Negl Trop Dis. 2015 Apr 1;9(4):e0003455. doi: 10.1371/journal.pntd.0003455 (PMC4382292; doi:10.1371/journal.pntd.0003455)
Supplement: S3 Fig — Mutations are indicated and the corresponding premature stop codon is identicated as underlined when occurring. The sequences are compared to that of the B. anthracis Ames strain. (DOC) [file pntd.0003455.s005.doc]

**Supplementary Figure 3:** *atxA* sequence alignment in CAR20 and CAR/CARP mutants

|  |  | 10 20 30 40 50 60 70 80 90 100 |
| --- | --- | --- |
|  |  | ....|....|....|....|....|....|....|....|....|....|....|....|....|....|....|....|....|....|....|....| |
|  | CAR | atgctaacaccgatatccatcgaaaaggaacatataagattaattaatttactacactttatcaatgaacaaaatagatggtttacaattaaagaattat |
|  | CAR1 | .................................................................................................... |
|  | CAR2 | ...............c.................................................................................... |
|  | CAR3 | .................................................................................................... |
|  | CAR4 | .................................................................................................... |
|  | CAR5 | .................................................................................................... |
|  | CAR20 | .................................................................................................... |
|  | CARP1 | .................................................................................................... |
|  | CARP2 | .................................................................................................... |
|  | CARP3 | .................................................................................................... |
|  | CARP5 | .................................................................................................... |
|  | CARP6 | .................................................................................................... |
|  | CARP7 | .................................................................................................... |
|  | CARP8 | .................................................................................................... |
|  | CARP12 | .................................................................................................... |
|  | BA_ames | .................................................................................................... |
|  |  | 110 120 130 140 150 160 170 180 190 200 |
|  |  | ....|....|....|....|....|....|....|....|....|....|....|....|....|....|....|....|....|....|....|....| |
|  | CAR | ctgattatctacaggtcgcagataaaacagtccgaaaatatttaaaattattagaagacg-aaattcctccatcttggaatttacttgttcaaaaaggaa |
|  | CAR1 | ............................................................-....................................... |
|  | CAR2 | ............................................................-....................................... |
|  | CAR3 | ............................................................-....................................... |
|  | CAR4 | ............................................................-....................................... |
|  | CAR5 | ............................................................-....................................... |
|  | CAR20 | ....................................................T...C.AG**T**....................................... |
|  | CARP1 | ............................................................-....................................... |
|  | CARP2 | ............................................................-....................................... |
|  | CARP3 | ............................................................-....................................... |
|  | CARP5 | ............................................................-....................................... |
|  | CARP6 | ............................................................-....................................... |
|  | CARP7 | ............................................................-....................................... |
|  | CARP8 | ............................................................-....................................... |
|  | CARP12 | ............................................................-....................................... |
|  | BA_ames | ............................................................-....................................... |
|  |  | 210 220 230 240 250 260 270 280 290 300 |
|  |  | ....|....|....|....|....|....|....|....|....|....|....|....|....|....|....|....|....|....|....|....| |
|  | CAR | aaggaatttatctaaaaaaaccattaaatgaatccctttcctttgttgaatcaaaaattttaagaaaatcactaaatcttcaaatttgcgaagaacttgt |
|  | CAR1 | .................................................................................................... |
|  | CAR2 | .................................................................................................... |
|  | CAR3 | .................................................................................................... |
|  | CAR4 | .....................a.............................................................................. |
|  | CAR5 | .................................................................................................... |
|  | CAR20 | ............................tga..................................................................... |
|  | CARP1 | .................................................................................................... |
|  | CARP2 | .................................................................................................... |
|  | CARP3 | .................................................................................................... |
|  | CARP5 | .................................................................................................... |
|  | CARP6 | .................................................................................................... |
|  | CARP7 | .................................................................................................... |
|  | CARP8 | .................................................................................................... |
|  | CARP12 | .................................................................................................... |
|  | BA_ames | .................................................................................................... |
|  |  |  |
|  |  |  |
|  |  | 310 320 330 340 350 360 370 380 390 400 |
|  |  | ....|....|....|....|....|....|....|....|....|....|....|....|....|....|....|....|....|....|....|....| |
|  | CAR | attcaaaaaaaa-cagtatgcaatccttagctcaaaaactccacttacaagtaggagctttatacccaattattaatcaaataaattatgatatacaatc |
|  | CAR1 | ............-....................................................................................... |
|  | CAR2 | ............-....................................................................................... |
|  | CAR3 | ............-....................................................................................... |
|  | CAR4 | ............-....................................................................................... |
|  | CAR5 | ............-....................................................................................... |
|  | CAR20 | ............-....................................................................................... |
|  | CARP1 | ............-....................................................................................... |
|  | CARP2 | ............a............................................................taa........................ |
|  | CARP3 | ............-....................................................................................... |
|  | CARP5 | ............-....................................................................................... |
|  | CARP6 | ............-....................................................................................... |
|  | CARP7 | ............-....................................................................................... |
|  | CARP8 | ............-....................................................................................... |
|  | CARP12 | ............-....................................................................................... |
|  | BA_ames | ............-....................................................................................... |
|  |  | 410 420 430 440 450 460 470 480 490 500 |
|  |  | ....|....|....|....|....|....|....|....|....|....|....|....|....|....|....|....|....|....|....|....| |
|  | CAR | cagtcatttaaatatcaaaaaaaaa-cctctagaaatatcgggaagagaacaagatgtccgcgtatttatgttaaggttatattgcaatattccaaatga |
|  | CAR1 | .........................-.......................................................................... |
|  | CAR2 | .........................-.......................................................................... |
|  | CAR3 | ........................--..............................................taa......................... |
|  | CAR4 | .........................-.......................................................................... |
|  | CAR5 | .........................-.......................................................................... |
|  | CAR20 | .........................-.......................................................................... |
|  | CARP1 | .........................-.......................................................................... |
|  | CARP2 | .........................-.......................................................................... |
|  | CARP3 | .........................-.......................................................................... |
|  | CARP5 | ........................--..............................................taa......................... |
|  | CARP6 | ........................--..............................................taa......................... |
|  | CARP7 | .........................**a**.......................................................................... |
|  | CARP8 | .........................-.......................................................................... |
|  | CARP12 | .........................**a**.......................................................................... |
|  | BA_ames | .........................-.......................................................................... |
|  |  |  |
|  |  |  |
|  |  | 510 520 530 540 550 560 570 580 590 600 |
|  |  | ....|....|....|....|....|....|....|....|....|....|....|....|....|....|....|....|....|....|....|....| |
|  | CAR | ttattggccgtttccctatattaataaacaaaatatcactgatttaattaataaaatggaaaaaaatttaaatgtacaaatgtacacctattcaaaacac |
|  | CAR1 | .................................................................................................... |
|  | CAR2 | .................................................................................................... |
|  | CAR3 | .................................................................................................... |
|  | CAR4 | .................................................................................................... |
|  | CAR5 | .................................................................................................... |
|  | CAR20 | .................................................................................................... |
|  | CARP1 | .................................................................................................... |
|  | CARP2 | .................................................................................................... |
|  | CARP3 | .................................................................................................... |
|  | CARP5 | .................................................................................................... |
|  | CARP6 | .................................................................................................... |
|  | CARP7 | .....................taa............................................................................ |
|  | CARP8 | .................................................................................................... |
|  | CARP12 | .....................taa............................................................................ |
|  | BA_ames | .................................................................t.................................. |
|  |  | 610 620 630 640 650 660 670 680 690 700 |
|  |  | ....|....|....|....|....|....|....|....|....|....|....|....|....|....|....|....|....|....|....|....| |
|  | CAR | aaattgtgtgtgttgttcgctataacaatctccagattactatcaggaaatacaatagataatgtaagtggacttattttagtaaataaaaatgatgatc |
|  | CAR1 | .................................................................................................... |
|  | CAR2 | .................................................................................................... |
|  | CAR3 | .................................................................................................... |
|  | CAR4 | .................................................................................................... |
|  | CAR5 | .................................................................................................... |
|  | CAR20 | .................................................................................................... |
|  | CARP1 | .................................................................................................... |
|  | CARP2 | .................................................................................................... |
|  | CARP3 | .................................................................................................... |
|  | CARP5 | .................................................................................................... |
|  | CARP6 | .................................................................................................... |
|  | CARP7 | .................................................................................................... |
|  | CARP8 | .................................................................................................... |
|  | CARP12 | .................................................................................................... |
|  | BA_ames | .................................................................................................... |
|  |  |  |
|  |  | 710 720 730 740 750 760 770 780 790 800 |
|  |  | ....|....|....|....|....|....|....|....|....|....|....|....|....|....|....|....|....|....|....|....| |
|  | CAR | attataaaacagtagcgtctataacctcagagctacaaaactctttcggcgttacattacatgaaactgagatttcatttttagcccttgcactactttt |
|  | CAR1 | .................................................................................................... |
|  | CAR2 | .................................................................................................... |
|  | CAR3 | .................................................................................................... |
|  | CAR4 | .................................................................................................... |
|  | CAR5 | .................................................................................................... |
|  | CAR20 | .................................................................................................... |
|  | CARP1 | .................................................................................................... |
|  | CARP2 | .................................................................................................... |
|  | CARP3 | .................................................................................................... |
|  | CARP5 | .................................................................................................... |
|  | CARP6 | .................................................................................................... |
|  | CARP7 | .................................................................................................... |
|  | CARP8 | .................................................................................................... |
|  | CARP12 | .................................................................................................... |
|  | BA_ames | .................................................................................................... |
|  |  |  |
|  |  | 810 820 830 840 850 860 870 880 890 900 |
|  |  | ....|....|....|....|....|....|....|....|....|....|....|....|....|....|....|....|....|....|....|....| |
|  | CAR | atctcttggaaattctattaccacagacagcaataaaacattaacttcctacaaaaaaa-caattatgcctttggctaaagaaattaccaaaggaattga |
|  | CAR1 | ...........................................................-........................................ |
|  | CAR2 | ...........................................................-........................................ |
|  | CAR3 | ...........................................................-........................................ |
|  | CAR4 | ...........................................................-........................................ |
|  | CAR5 | ...........................................................-........................................ |
|  | CAR20 | ...........................................................-........................................ |
|  | CARP1 | ...........................................................-........................................ |
|  | CARP2 | ...........................................................-........................................ |
|  | CARP3 | ...........................................................A................taa..................... |
|  | CARP5 | ...........................................................-........................................ |
|  | CARP6 | ...........................................................-........................................ |
|  | CARP7 | ...........................................................-........................................ |
|  | CARP8 | ...........................................................-........................................ |
|  | CARP12 | ...........................................................-........................................ |
|  | BA_ames | ...........................................................-........................................ |
|  |  |  |
|  |  | 910 920 930 940 950 960 970 980 990 1000 |
|  |  | ....|....|....|....|....|....|....|....|....|....|....|....|....|....|....|....|....|....|....|....| |
|  | CAR | acataaattacaacttgggataaattatgatgaatcttttttaacatatgttgttctaatcataaaaaaa-gcattagataaaaactttattcaatatta |
|  | CAR1 | ......................................................................-............................. |
|  | CAR2 | ......................................................................-............................. |
|  | CAR3 | ......................................................................-............................. |
|  | CAR4 | ......................................................................-............................. |
|  | CAR5 | ......................................................................-............................. |
|  | CAR20 | ......................................................................-............................. |
|  | CARP1 | ......................................................................A........taa.................. |
|  | CARP2 | ......................................................................-............................. |
|  | CARP3 | ......................................................................-............................. |
|  | CARP5 | ......................................................................-............................. |
|  | CARP6 | ......................................................................-............................. |
|  | CARP7 | ......................................................................-............................. |
|  | CARP8 | ......................................................................-............................. |
|  | CARP12 | ......................................................................-............................. |
|  | BA_ames | ......................................................................-............................. |
|  |  |  |
|  |  | 1010 1020 1030 1040 1050 1060 1070 1080 1090 1100 |
|  |  | ....|....|....|....|....|....|....|....|....|....|....|....|....|....|....|....|....|....|....|....| |
|  | CAR | taattataat------ataaaatttataaggcatataaaacagcgtcatccaaatacatttaatacaattcaagaatgcattagtaatttaaactataca |
|  | CAR1 | ....------------.................................................................................... |
|  | CAR2 | ..........------.................................................................................... |
|  | CAR3 | ..........------.................................................................................... |
|  | CAR4 | ..........------.................................................................................... |
|  | CAR5 | ....------------.................................................................................... |
|  | CAR20 | ..........------.................................................................................... |
|  | CARP1 | ..........------.................................................................................... |
|  | CARP2 | ..........------.................................................................................... |
|  | CARP3 | ..........------.................................................................................... |
|  | CARP5 | ..........------.................................................................................... |
|  | CARP6 | ..........------.................................................................................... |
|  | CARP7 | ..........------.................................................................................... |
|  | CARP8 | ..........Tataat.................................................................................... |
|  | CARP12 | ..........------.................................................................................... |
|  | BA_ames | ..........------.................................................................................... |
|  |  |  |
|  |  | 1110 1120 1130 1140 1150 1160 1170 1180 1190 1200 |
|  |  | ....|....|....|....|....|....|....|....|....|....|....|....|....|....|....|....|....|....|....|....| |
|  | CAR | gtatactcccatttcgactgctatgaaatttcattattaacaatgcattttgaaactcaacgtatgctatttaaaaataacccgaaaaaaatatatgtat |
|  | CAR1 | .................................................................................................... |
|  | CAR2 | .................................................................................................... |
|  | CAR3 | .................................................................................................... |
|  | CAR4 | .................................................................................................... |
|  | CAR5 | .................................................................................................... |
|  | CAR20 | .................................................................................................... |
|  | CARP1 | .................................................................................................... |
|  | CARP2 | .................................................................................................... |
|  | CARP3 | .................................................................................................... |
|  | CARP5 | .................................................................................................... |
|  | CARP6 | .................................................................................................... |
|  | CARP7 | .................................................................................................... |
|  | CARP8 | .................................................................................................... |
|  | CARP12 | .................................................................................................... |
|  | BA_ames | .................................................................................................... |
|  |  |  |
|  |  | 1210 1220 1230 1240 1250 1260 1270 1280 1290 1300 |
|  |  | ....|....|....|....|....|....|....|....|....|....|....|....|....|....|....|....|....|....|....|....| |
|  | CAR | acacctcacaaggatgtatacatcgagagtatatatctgcactgcttgaaaaacgttataatggacttattaaaattgtaagaaacactattataaattt |
|  | CAR1 | .................................................................................................... |
|  | CAR2 | .................................................................................................... |
|  | CAR3 | .................................................................................................... |
|  | CAR4 | .................................................................................................... |
|  | CAR5 | .................................................................................................... |
|  | CAR20 | .................................................................................................... |
|  | CARP1 | .................................................................................................... |
|  | CARP2 | .................................................................................................... |
|  | CARP3 | .................................................................................................... |
|  | CARP5 | .................................................................................................... |
|  | CARP6 | .................................................................................................... |
|  | CARP7 | .................................................................................................... |
|  | CARP8 | .................................................................................................... |
|  | CARP12 | .................................................................................................... |
|  | BA_ames | .................................................................................................... |
|  |  |  |
|  |  | 1310 1320 1330 1340 1350 1360 1370 1380 1390 1400 |
|  |  | ....|....|....|....|....|....|....|....|....|....|....|....|....|....|....|....|....|....|....|....| |
|  | CAR | aactaacgaatcactccaagacatggagatagatattattatttctaatgttaacttacctataaaaaatatacctattgtacaaatttcggaatttcct |
|  | CAR1 | .................................................................................................... |
|  | CAR2 | .................................................................................................... |
|  | CAR3 | .................................................................................................... |
|  | CAR4 | .................................................................................................... |
|  | CAR5 | .................................................................................................... |
|  | CAR20 | .................................................................................................... |
|  | CARP1 | .................................................................................................... |
|  | CARP2 | .................................................................................................... |
|  | CARP3 | .................................................................................................... |
|  | CARP5 | .................................................................................................... |
|  | CARP6 | .................................................................................................... |
|  | CARP7 | .................................................................................................... |
|  | CARP8 | .................................................................................................... |
|  | CARP12 | .................................................................................................... |
|  | BA_ames | .................................................................................................... |
|  |  |  |
|  |  | 1410 1420 1430 |
|  |  | ....|....|....|....|....|....|....|.... |
|  | CAR | acagaaagagattttcatgaaatcaaaaagataataTAA |
|  | CAR1 | ....................................... |
|  | CAR2 | ....................................... |
|  | CAR3 | ....................................... |
|  | CAR4 | ....................................... |
|  | CAR5 | ....................................... |
|  | CAR20 | ....................................... |
|  | CARP1 | ....................................... |
|  | CARP2 | ....................................... |
|  | CARP3 | ....................................... |
|  | CARP5 | ....................................... |
|  | CARP6 | ....................................... |
|  | CARP7 | ....................................... |
|  | CARP8 | ....................................... |
|  | CARP12 | ....................................... |
|  | BA_ames | ....................................... |
